# Hypoxia‐Responsive Prodrug of ATR Inhibitor, AZD6738, Selectively Eradicates Treatment‐Resistant Cancer Cells

**DOI:** 10.1002/advs.202403831

**Published:** 2024-07-08

**Authors:** Francis M. Barnieh, Goreti Ribeiro Morais, Paul M. Loadman, Robert A. Falconer, Sherif F. El‐Khamisy

**Affiliations:** ^1^ Institute of Cancer Therapeutics Faculty of Life Sciences University of Bradford Richmond Road Bradford BD7 1DP United Kingdom; ^2^ School of Biosciences, the Healthy Lifespan Institute and the Institute of Neuroscience University of Sheffield Sheffield S10 2TN United Kingdom

**Keywords:** ATR, AZD6738, DNA repair, Hypoxia, Prodrugs, Targeted Delivery

## Abstract

Targeted therapy remains the future of anti‐cancer drug development, owing to the lack of specificity of current treatments which lead to damage in healthy normal tissues. ATR inhibitors have in recent times demonstrated promising clinical potential, and are currently being evaluated in the clinic. However, despite the considerable optimism for clinical success of these inhibitors, reports of associated normal tissues toxicities remain a concern and can compromise their utility. Here, ICT10336 is reported, a newly developed hypoxia‐responsive prodrug of ATR inhibitor, AZD6738, which is hypoxia‐activated and specifically releases AZD6738 only in hypoxic conditions, in vitro. This hypoxia‐selective release of AZD6738 inhibited ATR activation (T1989 and S428 phosphorylation) and subsequently abrogated HIF1a‐mediated adaptation of hypoxic cancers cells, thus selectively inducing cell death in 2D and 3D cancer models. Importantly, in normal tissues, ICT10336 is demonstrated to be metabolically stable and less toxic to normal cells than its active parent agent, AZD6738. In addition, ICT10336 exhibited a superior and efficient multicellular penetration ability in 3D tumor models, and selectively eradicated cells at the hypoxic core compared to AZD6738. In summary, the preclinical data demonstrate a new strategy of tumor‐targeted delivery of ATR inhibitors with significant potential of enhancing the therapeutic index.

## Graphical abstract

The hypoxic treatment‐resistant fraction of triple‐negative breast cancer is responsible for the deadly metastatic recurrence associated with the disease. This study reports an effective and non‐toxic therapeutic strategy that selectively eradicate hypoxic TNBC cells, by releasing ATR inhibitor only in hypoxic conditions.

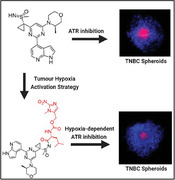

## 1 Introduction

Triple‐negative breast cancer (TNBC) is an aggressive subtype of breast cancer accounting for 15–25% of all breast cancers, and 24% of newly diagnosed cases.^[^ ^1^, ^2^ ^]^ Clinical TNBCs lack the expression of oestrogen (ER), progesterone (PR), and HER2 receptors, which means patients are not suitable for either hormone or anti‐HER‐2 therapy. Compared to other subtypes of breast cancer, TNBC patients have worse clinical outcomes after treatment (usually neoadjuvant chemotherapy) with higher recurrence rates.^[^ ^3^, ^4^ ^]^ Thus, there is a current unmet need for a better treatment options for patients. With that said, recent advances in DNA damage repair (DDR) inhibitors in TNBC treatment hold significant promise.^[^ ^5^ ^]^ The DDR machinery comprises the mechanisms through which DNA damage is detected and repaired to maintain genomic stability and integrity. This is critical to the survival of cells, but more importantly to cancer cells.^[^ ^6^ ^]^ Loss or defective DDR components are a common feature with TNBC, which often leads to a greater reliance of cancer cells on compensatory residual DDR factors for survival.^[^ ^7^, ^8^, ^9^ ^]^ Consequently, DDR mechanisms have been of notable interest to the treatment of TNBC with inhibitors of DDR key players ATR (Ataxia Telangiectasia Mutated and Rad‐3 Related protein kinase), DNA‐PK (DNA‐dependent protein kinase), and WEE1 currently being investigated in both preclinical and clinical TNBC.^[^ ^5^, ^10^, ^11^, ^12^ ^]^

ATR, an apical regulator of the DDR pathway, is one of the most studied DDR elements in TNBC with significant potential.^[^ ^5^, ^13^, ^14^, ^15^ ^]^ The ATR kinase initiates and coordinates repair of DNA damage associated with single‐strand DNA accumulation which may arise from resected DNA DSB, cross‐links, or base adduct and inhibition of DNA polymerases.^[^ ^16^ ^]^ The dependence of tumor cells on the ATR pathway, particularly in response to DNA damage is well‐established,^[^ ^7^, ^17^, ^18^ ^]^ with ATR inhibition demonstrated as an effective treatment for TNBCs in various preclinical TNBC models particularly as a chemo‐ or radio‐sensitizer.^[^ ^13^, ^14^, ^15^, ^19^, ^20^ ^]^ Several ATR inhibitors (ATRi) have now entered clinical evaluation in many solid advanced tumors including TNBC.^[^ ^21^, ^22^ ^]^ One such clinical candidate is AZD6738 (ceralasertib),^[^ ^23^ ^]^ which is currently undergoing evaluation in clinical trials for treatment of TNBC or metastatic breast cancer. While the results of these clinical trials are eagerly awaited, reported normal tissue toxicities associated with ATR inhibition both in pre‐clinical and clinical settings remain a concern.^[^ ^19^, ^24^, ^25^, ^26^, ^27^ ^]^ Considering that ATR is an essential gene, with its loss leading to embryonic lethality,^[^ ^28^, ^29^ ^]^ and its recently reported anti‐apoptotic role in normal cells,^[^ ^30^ ^]^ the clinical utility of ATRi is likely to be compromised due to systemic toxicity, despite its promising clinical potential.^[^ ^21^ ^]^ Hence, the need for a targeted approach of ATRi delivery into tumors.

ATR, despite its established activation with DNA damage, is now known to be activated in tumor hypoxia even in the absence of DNA damage.^[^ ^31^ ^]^ Hypoxia activation of ATR in tumors is critical for cellular adaptation to hypoxia,^[^ ^32^ ^]^ thus promoting tumor angiogenesis,^[^ ^33^ ^]^ cell motility and invasion,^[^ ^34^ ^]^ and survival.^[^ ^35^, ^36^ ^]^ Subsequent to these observations, ATR and its activation have been suggested as a potential therapeutic target for eradicating hypoxic cancer cells.^[^ ^31^, ^37^ ^]^ Tumor hypoxia, a condition in which tumor cells are deprived of adequate oxygen supply, is a major feature of TNBCs and strongly linked to the chemoresistance and poor prognosis of the disease.^[^ ^38^, ^39^ ^]^ Considering the hypoxic nature of clinical TNBC compared to most normal tissues and the demonstrated critical function of ATR in hypoxia adaptation of cancer cells independent of its DDR function, we suggest that hypoxia‐activated release of an ATRi (AZD6738) represents a favorable strategy for selectively targeting ATR function in TNBC whilst sparing normal cells (reduced toxicity).

It is worth stating many hypoxia activated prodrugs (HAP), which predominantly focused on the release of a cytotoxic agent in response to hypoxia have been previously reported, although none with clinical success.^[^ ^40^, ^41^, ^42^ ^]^ Of note, these cytotoxic agents lack hypoxia‐specific effects and do not molecularly target the hypoxic microenvironment.^[^ ^43^ ^]^ Contrary to the previous reported approach, we describe a strategy to release a molecularly‐targeted inhibitor of a protein (ATR), whose functions are emerging to be critical for the adaptation and survival of treatment‐resistant hypoxic cancer cells. This is a more beneficial and non‐toxic approach in eradicating hypoxic TNBC cells, and in addition provides a local bystander effect through diffusion of the active agent on neighboring non‐hypoxic TNBC cells.

**Scheme 1:**
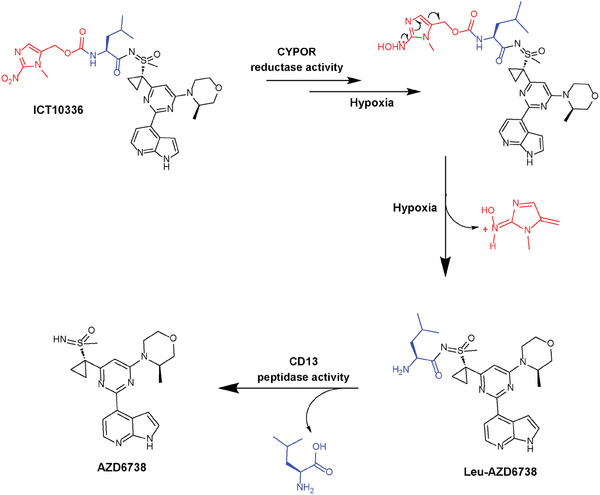
Structure of ICT10336 and proposed hypoxia‐dependent metabolism to release active agent AZD6738 within hypoxic tumor microenvironment.

As a proof‐of‐concept, we have explored this strategy with the development of nitroaromatic hypoxia‐activated prodrugs of AZD6738, which are reduced by NADPH‐cytochrome P450 oxidoreductase (CYPOR) and subsequently metabolized by aminopeptidase CD13 to release AZD6738 in the hypoxic tumor microenvironment (**Scheme** **1**). High expression of CYPOR is an independent prognostic biomarker of poor survival of TNBC patients,^[^ ^44^ ^]^ and high activity of CD13 in tumors is well‐documented.^[^ ^45^ ^]^ We have demonstrated that the developed AZD6738‐prodrug, ICT10336, is hypoxia‐specific and activated by CYPOR and CD13 to release AZD6738 (ATRi), which blocks HIF1α‐mediated hypoxia adaptation and selectively induces DNA damage in hypoxic TNBC. ICT10336 was demonstrated to be less toxic to normal cells compared to the DNA damage and toxicity observed with its active parent agent, AZD6738. In addition, in 3D TNBC models ICT10336 demonstrated an efficient multicellular penetration ability and abrogated HIF‐1A mediated responses, thus induces cells death.

## 2 Results

### 2.1 Hypoxia Sensitises Triple‐Negative Breast Cancer Cells to AZD6738

**Figure 1:**
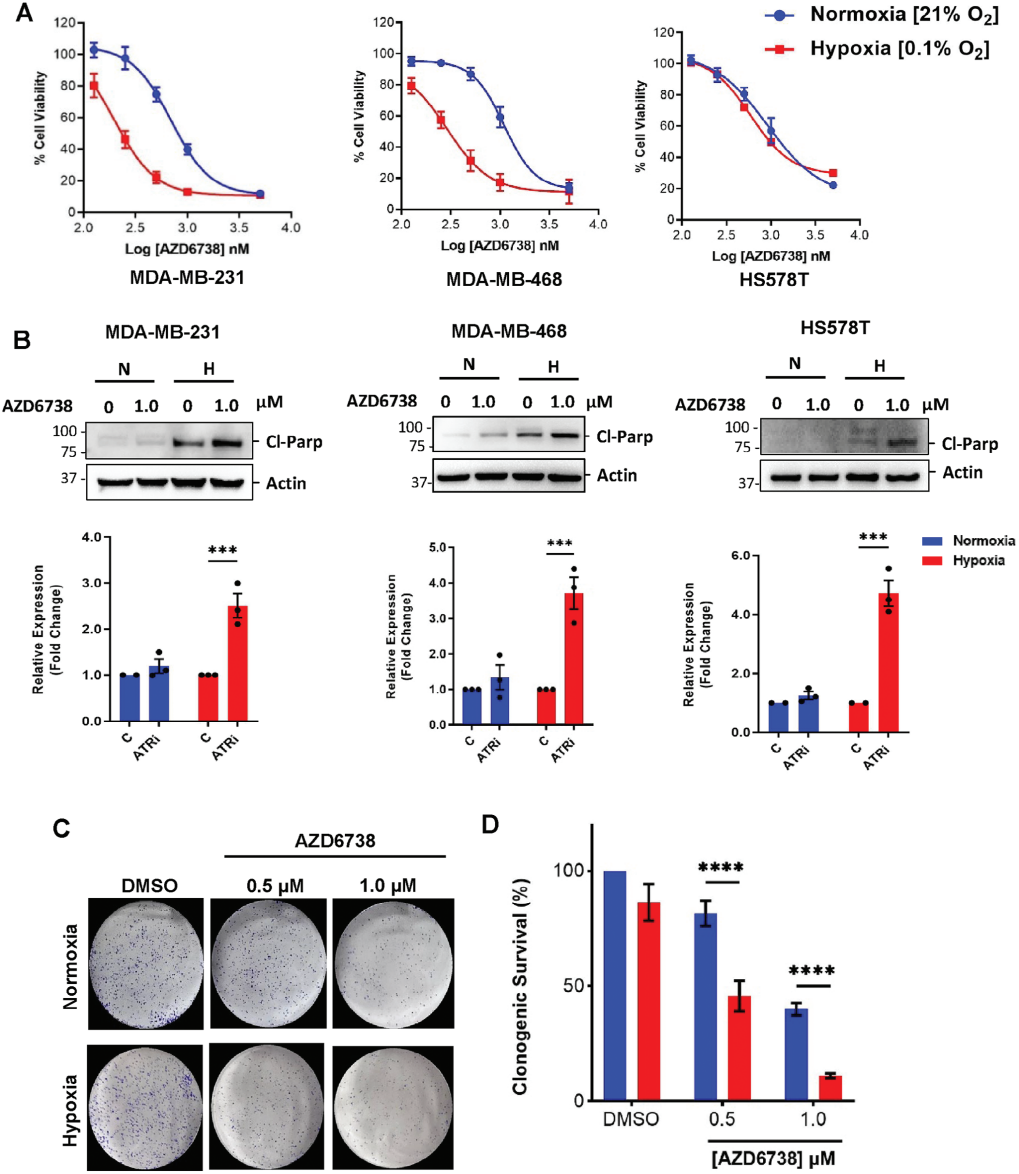
TNBC is sensitive to AZD6738 in hypoxic conditions compared to normoxic conditions. A) TNBC cells were treated with various concentrations of AZD6738 in normoxic and hypoxic conditions, for 96 h and cell viability was assessed via an MTT assay. B) TNBC cell treated with AZD6738 (1 µm) for 24 h in either normoxic (N) or hypoxic (H) conditions and cell lysates were analyzed by immunoblotting with anti‐ cleaved PARP. C,D) Clonogenic assay results after exposure of MDA‐MB‐468 cells to AZD6738 for 24 h in normoxic and hypoxic conditions followed by regrowth in drug‐free medium in normoxic conditions for 12 days; Representative images of crystal violet stained colonies (C), and relative quantification of the stained colonies in each treatment using ImageJ (D). Data shown are the mean of ≥ 3 independent experiments ± SEM. ***p > 0.001 and ****p > 0.0001 (two‐way ANOVA).

Hypoxia‐dependent activation of the ATR signaling pathway has been recently reported in hypoxic cancer cells.^[^ ^31^ ^]^ We first determined whether hypoxia sensitize TNBC cells to AZD6738, an ATR inhibitor. To achieve this, we treated 3 different TNBC cells (MDA‐MB‐231, MDA‐MB‐468 and Hs578T) with AZD6738 in either normoxic (21% O_2_) or hypoxic (0.1% O_2_) conditions, either continuously for 96 h, or for 24 h followed by 72 h regrowth in drug‐free medium in normoxic conditions. The sensitivity of hypoxic TNBC cells (MDA‐MB‐231 and MDA‐MB‐468) to AZD6738 significantly increased (two‐ to four‐fold) compared to normoxic controls, in both treatment conditions. Interestingly, this was not the case for Hs578T cells, however (**Figure** **1**A and **Table** **1**; Figure S1A, Supporting Information). The observed increased chemosensitivity to AZD6738 in hypoxic TNBC cells correlated to increased cleaved PARP expression in these cells, indicating an increased level of apoptosis (Figure 1B). Surprisingly, AZD6738 treatment of Hs578T cells in hypoxia led to a significant increase in cleaved PARP compared to normoxic controls, despite the observed lack of change in chemosensitivity. In addition, using a colony survival assay, the regrowth ability of TNBC cells after 24 h AZD6738 treatment in hypoxia was significantly reduced compared to normoxic controls (Figure 1C,D; Figure S1B, Supporting Information). This data demonstrates the sensitivity of hypoxic TNBC to ATR inhibition and suggests a potential superior benefit of AZD6738 in hypoxic TNBC cells compared to normoxic TNBC cells.

**Table 1:** IC_50_ values of AZD6738 in TNBC (A) after 96h treatment in normoxic or hypoxia, and (B) after 24 h treatment in normoxia or hypoxia and 72 h regrowth in drug‐free medium in normoxic conditions.

TNBC	IC_50_ [µm]		HCR
	Normoxia	Hypoxia	
A)			
MDA‐MB‐231	0.75 ± 0.05	0.23 ± 0.03	**3.3**
MDA‐MB‐468	1.15 ± 0.28	0.28 ± 0.02	**4.1**
HS‐578T	1.21 ± 0.22	1.05 ± 0.07	**1.2**
B)			
MDA‐MB‐231	1.33 ± 0.39	0.78 ± 0.05	**1.8**
MDA‐MB‐468	2.12 ± 0.19	0.71 ± 0.08	**3.0**
HS‐578T	2.63 ± 0.38	2.20 ± 0.44	**1.2**

Hypoxia Cytotoxicity Ratio (HCR) is the ratio of the cytotoxicity (IC_50_) of the AZD6738 observed in normoxic conditions to the cytotoxicity (IC_50_) observed in hypoxic conditions.

### 2.2 AZD6738 Inhibits HIF‐ α Adaptation and Induces DNA Damage in Hypoxic TNBC

**Figure 2:**
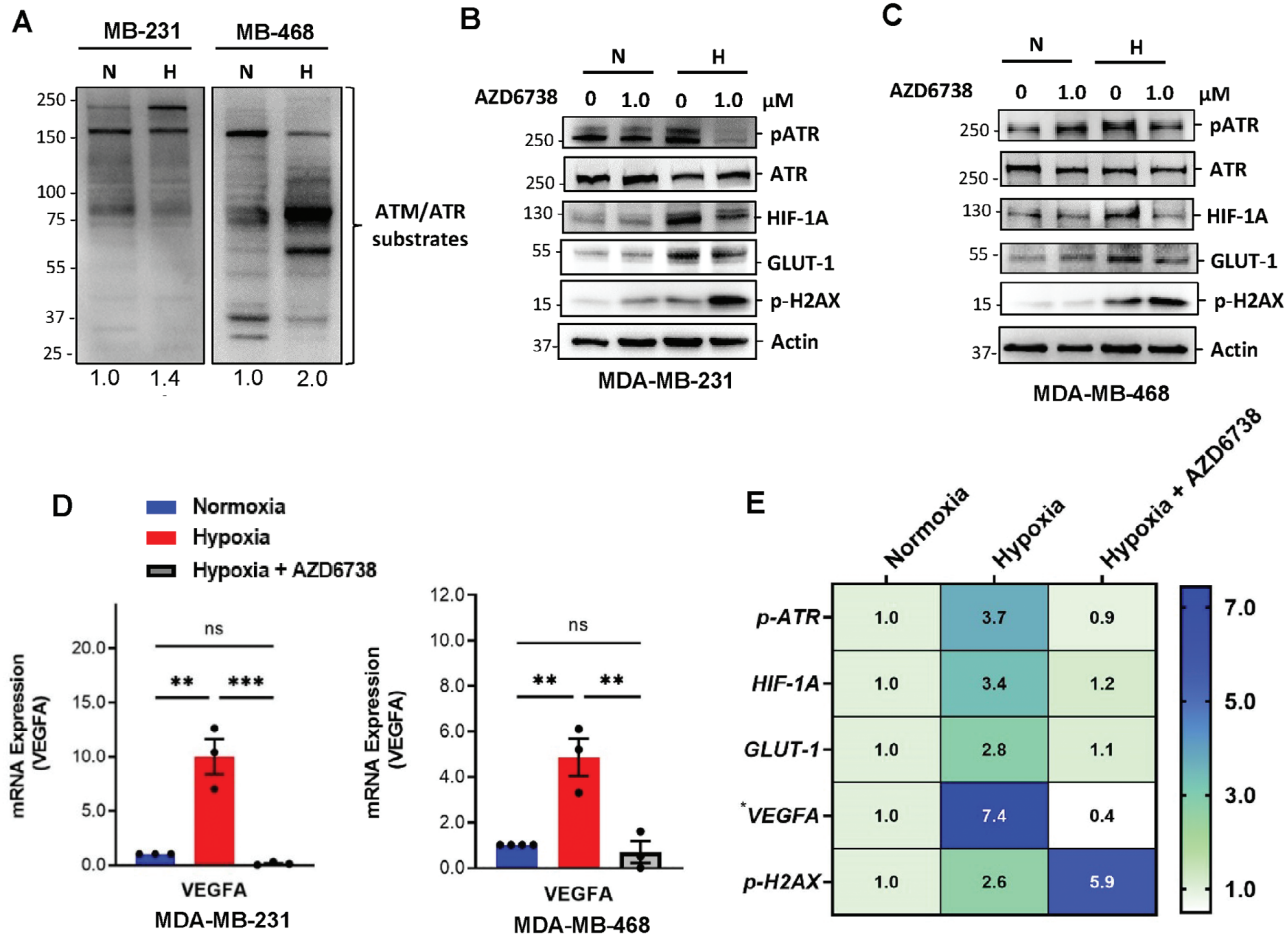
AZD6738 abrogates TNBC cell adaptive mechanisms and induces DNA damage in a hypoxic microenvironment. A) Hypoxia‐dependent phosphorylation of ATM/ATR protein substrates of TNBC cells after 6 h using pan phospho‐ATM/ATR antibody. MDA‐MB‐231 cells B) and MDA‐MB‐468 cells C) were treated with AZD6738 (1.0 µm) in hypoxic and normoxic conditions, and lysates immunoblotted for p‐ATR, ATR, HIF‐1A, GLUT‐1, and p‐H2AX expression. Band intensity was measured by Image Lab Software 6.1 and normalized to a β‐actin loading; D) AZD6738 inhibits hypoxia‐induced VEGF mRNA expression in MDA‐MB‐231 cells and MDA‐MB‐468 cells; E) Heat map of quantified mean relative expression of p‐ATR, HIF‐1A, GLUT‐1, and p‐H2AX (band intensities in Figure 2B,C; Figure S1C, Supporting Information) and VEGF mRNA expression in the presence and absence of AZD6738 (1.0 µm) in hypoxic TNBC cells (MDA‐MB‐231, MDA‐MB‐468, and HS578T). Data shown are the mean of ≥ 3 independent experiments ± SEM. *ns – not significant*, **p > 0.01, and ***p > 0.001 (two‐way ANOVA). N = normoxia; H = hypoxia.

Next, we investigated the molecular mechanism underlying the observed sensitivity of hypoxic TNBC to AZD6738. Hypoxia increases the phosphorylation of ATR/ATM substrates in TNBC cells (**Figure** **2**A). This was done using pan phospho‐ATM/ATR substrate antibody. Previous reports suggest that ATR activation is critical for HIF‐1A dependent cellular adaptation to hypoxia.^[^ ^32^, ^33^ ^]^ To test this, we treated 3 different TNBC cell lines with AZD6738 in hypoxia for 6 hours and determined its effects on the HIF‐1A mediated hypoxia response. Consistent with reported observations, ATR was phosphorylated (S428) in response to hypoxia in all tested TNBC cells with observed stabilization and accumulation of HIF‐1A protein (Figure 2B,C). However, the presence of AZD6738 in hypoxic TNBC cells abrogated the accumulation of HIF‐1A protein, suggesting that AZD6738 interferes with the expression or stabilization of HIF‐1A in hypoxic TNBC cells (Figure 2B,C; Figure S1C, Supporting Information). This observation is consistent with reported ATR involvement in HIF‐1A regulation.^[^ ^32^ ^]^ HIF‐1A regulates the adaptive response of cells to hypoxia by modulating the transcription of various target genes.^[^ ^47^ ^]^ We therefore assessed the effect of AZD6738 on the expression of two HIF‐1 target genes; GLUT‐1 and VEGFA, both involved in cellular adaptation to hypoxia. In hypoxic TNBC cells, AZD6738 significantly inhibited the hypoxia‐induced increased expression of GLUT‐1 and VEGFA (Figure 2B–E). Consistent with HIF‐1A being a transcription regulator, the observed effects on GLUT‐1 and VEGFA protein expression was due to differences in their transcript levels (Figure 2B–E). Additionally, relatively high levels of gamma‐H2AX phosphorylation, a DNA damage marker, were observed in AZD6738‐treated hypoxic TNBC cells compared to AZD6738‐treated normoxic TNBC cells. This data indicates the potency of AZD6738 to disrupt the adaptive and survival mechanisms of hypoxic TNBC cells, and promote the selective induction and accumulation of DNA damage in the hypoxic microenvironment.

### 2.3 ATR Inhibition is Comparably Toxic to Normal Human Healthy Cells

**Figure 3:**
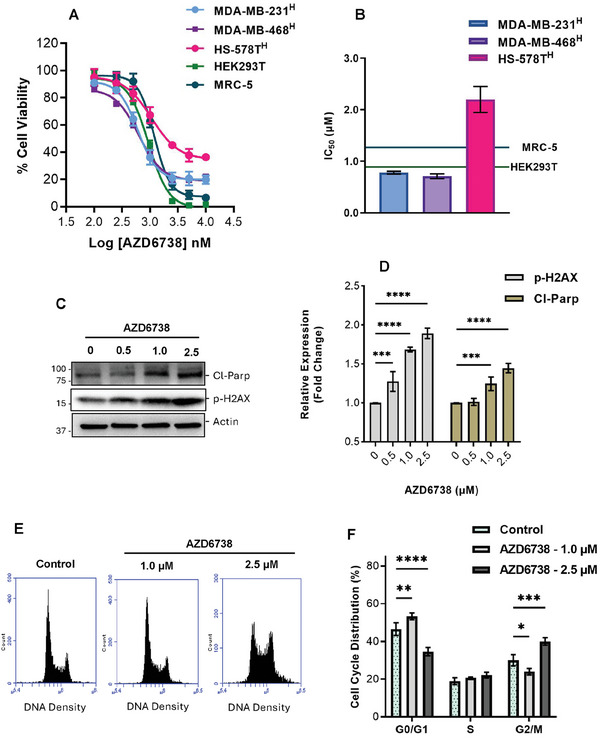
ATR inhibition induces DNA Damages accumulation and apoptosis in normal human cells. A) Hypoxic TNBC cells and normal human cells were treated with various concentrations of AZD6738 in normoxic and hypoxic conditions for 24 h followed by 72 h regrowth in drug‐free medium in normoxic conditions. Cell viability was assessed via an MTT assay. B) Comparable IC_50_ of AZD6738 in normal human cells and hypoxic TNBC cells. Normal human lung fibroblasts (MRC‐5) were treated with different concentrations of AZD6738 for 24 h. Lysates were analyzed for cleaved PARP and pH2AX expression: C) Western blot using anti‐ cleaved PARP and pH2AX antibodies and D) quantified relative expression cleaved PARP and pH2AX as detected by anti‐ cleaved PARP and pH2AX antibodies. Band intensity measured by Image Lab Software 6.1 and normalized to a β‐actin loading. AZD6738‐treated MRC‐5 cells were fixed with 66% ethanol and stained with propidium iodide. Cell cycle distributions of MRC‐5 were determined and quantified using flow cytometry E,F). Data shown are the mean of ≥ 3 independent experiments ± SEM. *p > 0.05, **p > 0.01, ***p > 0.001 and ****p > 0.0001 (two‐way ANOVA).

ATR is an essential gene and its inhibition has been reported to be toxic to normal cells.^[^ ^19^, ^26^, ^48^ ^]^ To corroborate this observation with AZD6738, we conducted chemosentitvity assays on two normal non‐cancerous human cell lines (MRC‐5 and HEK‐293T) with AZD6738 in normoxic conditions. We observed comparable or higher sensitivity of these normal cells to AZD6738 compared to hypoxic TNBCs (**Figure** **3**A,B). We further determined the level of apoptosis (cleaved PARP) and presence of DNA damage accumulation (p‐H2AX) in these normal cells after 24 h treatment with different concentrations of AZD6738. Significant p‐H2AX signal was observed at all tested AZD6738 concentrations (even at IC_10_ concentration in hypoxic TNBC cells) in normal human lung fibroblasts (MRC‐5), suggesting increased levels of DNA damage accumulation. This corresponded to an observed increase in cleaved PARP expression, indicating an increased level of apoptosis (Figure 3C,D). In addition, using cell cycle analysis, 24 h AZD6738 treatment was observed to alter the cell cycle profile of normal cells, thus inducing significant G0‐G1 arrest at 1.0 µm and G2/M arrest at 2.5 µm concentrations (Figure 3E,F). Together, this data reiterates the essentiality of ATR and demonstrates the potential toxic effects of AZD6738 to normal cells.

### 2.4 Compound Design and Synthesis

**Scheme 2:**
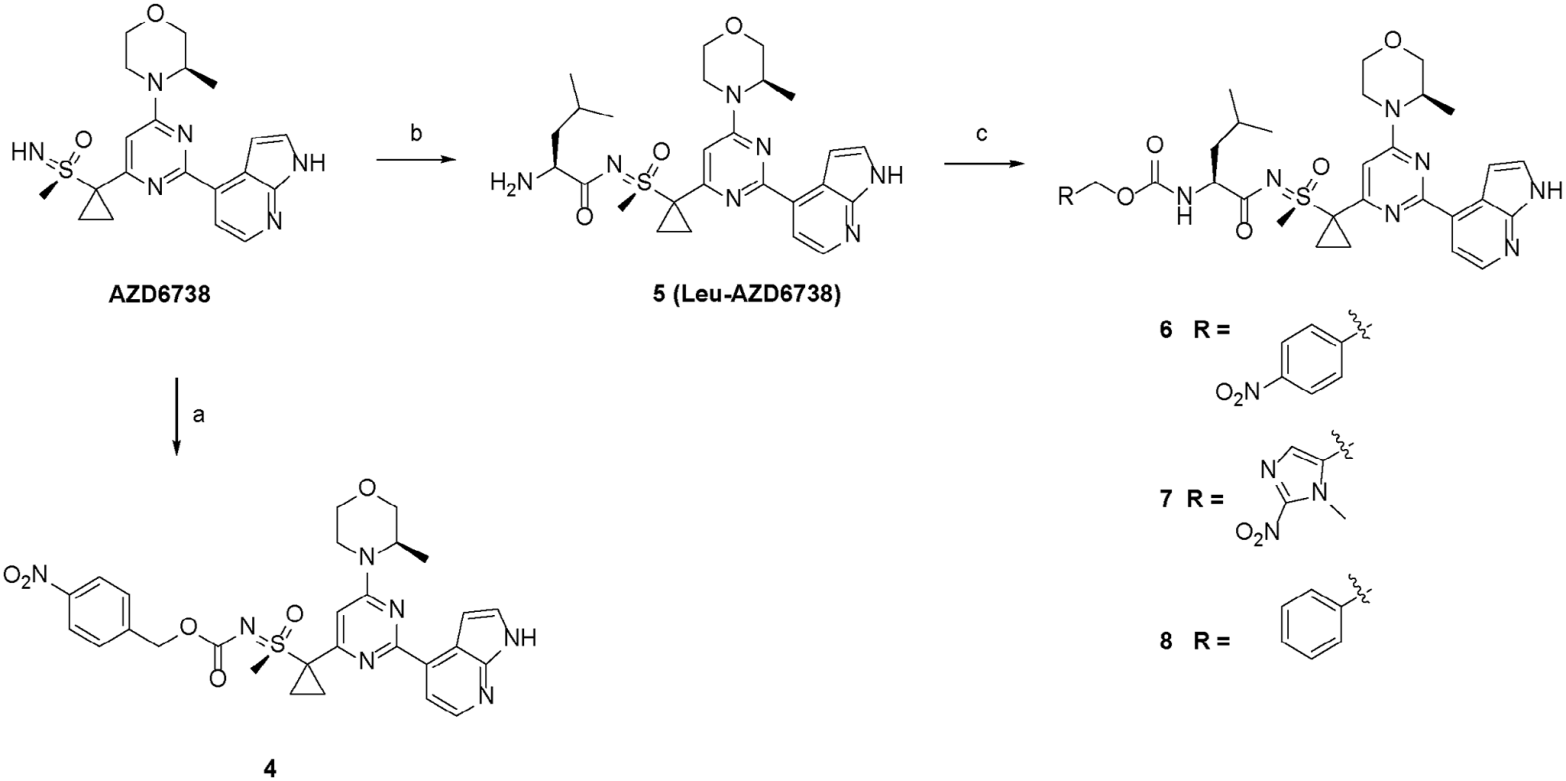
General synthesis of hypoxia activated AZD6738 analogues and negative control. *Reagents and conditions*: a) *p*‐nitrobenzyl chloroformate, pyridine, CH_2_Cl_2_; b) i. Fmoc‐Leu‐OH, Diisopropylcarbodiimide, CH_2_Cl_2_; ii. 20% piperidine in THF; c) DMAP, CH_2_Cl_2_ and intermediates **1**, **2**, and **3** (see Figure S4, Supporting Information) for compounds **6** (ICT10335), **7** (ICT10336) and **8** (ICT10337), respectively.

To selectively target the hypoxia‐dependent ATR functions in TNBCs whilst sparing its functions in normal cells, we developed a series of bioreductive prodrugs of AZD6738. The synthesis of the prodrugs was envisaged via carbamate formation between the reaction of the sulfoximine group of AZD6738 and a 4‐nitrophenyl carbonate intermediate (Figure S4, Supporting Information). Carbonate intermediates **1** and **3** were commercially obtained, while **2** was synthesized according to the literature.^[^ ^49^ ^]^ The carbamate group is expected to be easily hydrolyzed upon bioreduction of the nitro group to an amine, followed by spontaneous self‐immolation of the respective amino‐aryl/heteroaryl via 1,6‐elimination with release of carbon dioxide and AZD6738. Although the sulfoximine group of the AZD6738 is nucleophilic enough to react with activated carboxylic acids to form the respective *N*‐acylated analogues, this group was unreactive toward the carbonate group of compound **1**. The nitrogen indole was equally inert for the 4‐nitrophenyl carbonates. We also attempted the formation of 4‐nitrophenyl carbamate of AZD6738 followed by the reaction with *p*‐nitrobenzyl alcohol instead. In this case, conjugation of the formyl group with the sulfoximine renders a stable carbamate with reduced electrophilicity and not susceptible to be attacked by the alcohol. The *p*‐nitrobenzyl carbamate of AZD6738 could not be attained in this manner. Synthesis of carbamate prodrug **4** was then successfully achieved when AZD6738 was reacted with nitrobenzyl chloroformate instead of **1** (**Scheme** **2**), albeit in modest yield (51%). Both sulfoximine and indole groups were found to react with the more electrophilic chloro‐formyl group, leading to the formation of both regioisomer carbamates.

To overcome the observed challenges with the 4‐nitrophenyl carbonate synthesis, and limited commercial availability of the chloroformate intermediate of [(nitroimidazolyl)methanol], we designed an alternative approach by inserting a leucine residue between AZD6738 and the carbamate to furnish prodrugs **6** (ICT10335) and **7** (ICT10336). Diisopropylcarbodiimide‐mediated conjugation of the sulfoximine group of AZD6738 with Fmoc‐Leu‐OH, followed by hydrolysis of the Fmoc protective group afforded Leu‐AZD6738, which then successfully reacted with the carbonate intermediates presented in Figure S4 (Supporting Information), to obtain desired prodrugs **6** and **7** in good yields (70%) (Scheme 2). Negative control carbamate **8** (does not contain a bio‐reducible functional group) was assembled with the same strategy.

### 2.5 ICT10336 is Activated by *CYPOR* Enzymes under Hypoxic Conditions

**Figure 4:**
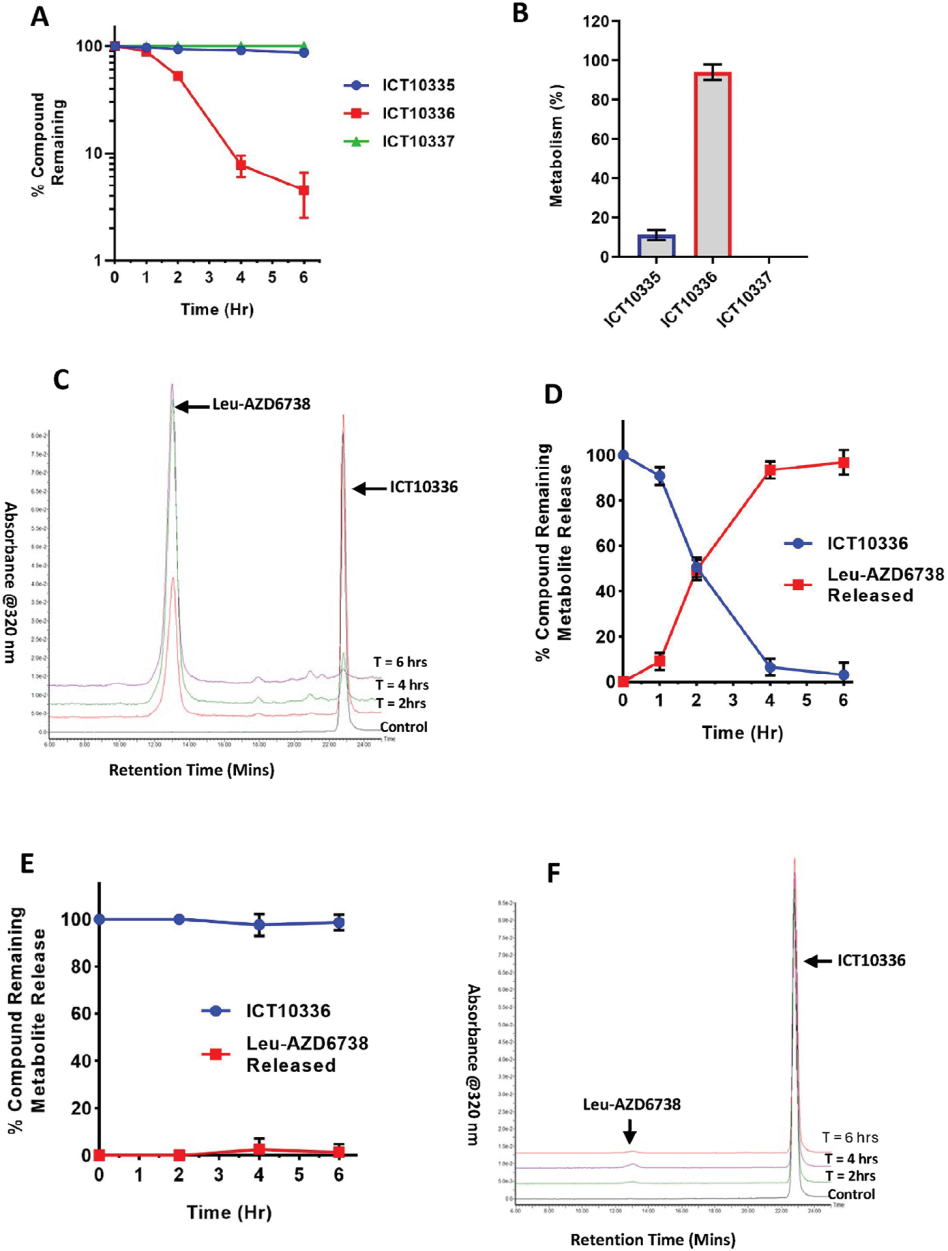
ICT10336 is reduced by *CYPOR* reductase in a hypoxia‐dependent manner. Nitroaryl‐based bioreductuve prodrugs of AZD6738 (10 µm) were incubated with bactosomal NADPH‐CYP reductase in hypoxic (0.1% O_2_) conditions for 6 h and monitored over incubation period using LC‐MS A), and calculated percentage of metabolism/bioreduction of prodrugs B). C) Representative of LC‐MS chromatogram of CYP‐reductase mediated metabolism of ICT10336, and D) quantified percentage of ICT10336 reduction and release of Leu‐AZD6738 over incubation period in hypoxic (0.1% O_2_) conditions. E) ICT10336 showed no bioreduction in normoxic conditions in the presence of CYP‐reductase. F) Representative LC‐MS chromatograms of CYP‐reductase mediated metabolism of ICT10336 over incubation period in normoxic (21% O_2_) conditions. Data shown are the mean of ≥ 2 independent experiments.

Nitroaryl‐based bioreductive prodrugs are known to be activated and reduced by CYP reductases (*CYPOR*) under hypoxic conditions.^[^ ^50^ ^]^ Hence, to assess bioreductive potential, the synthesised prodrugs were incubated with bactosomal human NADPH‐CYP reductase under both normoxic and hypoxic conditions for 6 hours, with AZD6738‐related metabolites monitored by LC‐MS.^[^ ^51^ ^]^ Under hypoxic conditions, both ICT10335 (4‐nitrobenzyl derivative) and ICT10336 (1‐methyl‐2‐nitroimidazole derivative) were bioreduced over the incubated time period, whilst the benzyl derivative lacking a nitro group (control compound ICT10337) led to no bioreduction (**Figure** **4**A,B). Bioreduction of ICT10336 was almost complete (95%) after 6 h of incubation leading to successful release of Leu‐AZD6738 (Figure 4C,D), whilst only 15% of ICT10335 was bioreduced. Crucially, when incubated with CYP reductases in normoxic condtions, ICT10336 demonstrated no bioreduction or release of Leu‐AZD6738 (Figure 4E,F), confirming the hypoxia dependent manner of ICT10336 bioreduction observed. Together, this data suggests ICT10336 as a hypoxia‐dependent *CYPOR* mediated bioreductive prodrug of AZD6738 as proposed. It is worth noting that the release of Leu‐AZD6738 rather than AZD6738 as observed was expected. Leu‐AZD6738 is expected to be metabolised by CD13 which was absent in this assay.

### 2.6 Hypoxia‐Dependent Activation and Metabolism of ICT10336 in Cancer Cells

**Figure 5:**
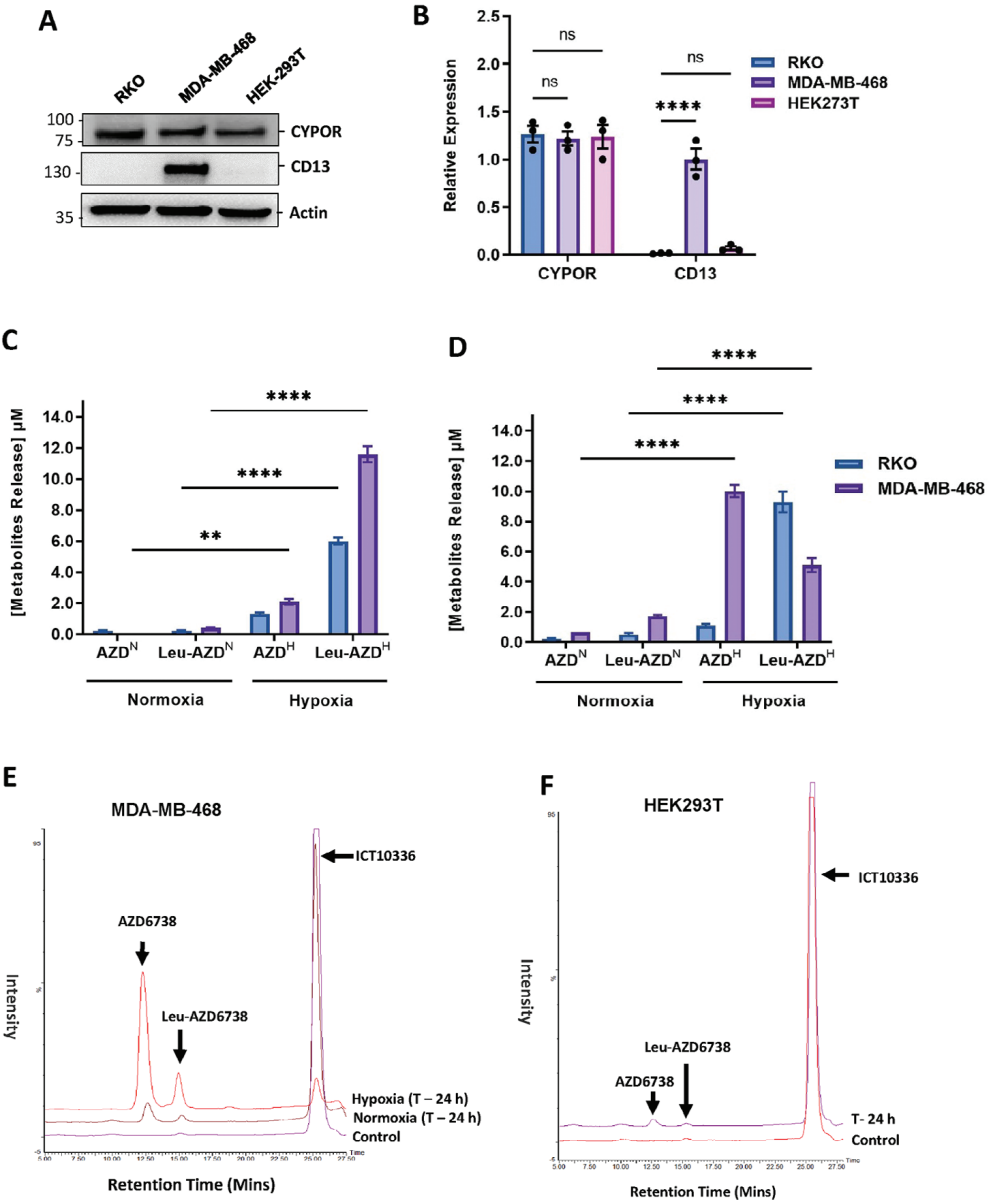
Cellular activation and release of AZD6738 from ICT10336 is hypoxic‐dependent. *CYPOR* protein expression in cancer cells, (MDA‐MB‐468 and RKO) and normal cells (HEK297T); A) Western blot using anti‐*CYPOR* antibody and B) quantified relative expression of *CYPOR* in cells as detected by anti‐*CYPOR* antibodies. Band intensity was measured by Image Lab Software 6.1 and normalized to a β‐actin loading. Quantified in vitro cellular (combined intracellular and extracellular) concentration of Leu‐AZD6738 and free AZD6738 following ICT10336 incubation with cancer cells for 6 h C) and 24 h D) in normoxic and hypoxic conditions. E) Representative of Selective Ion Recording (SIR) chromatogram demonstrating differential metabolism of ICT10336 in normoxic and hypoxic MDA‐MB‐468 cells. F) Representative Selective Ion Recording (SIR) chromatogram showing negligible ICT10336 metabolism and release of active agent (AZD6738) in normoxic normal human cells, HEK297T. Quantified data shown are the mean of ≥ 2 independent experiments.

Next, we examined the potential bioreduction and release of free AZD6738 from ICT10336 in hypoxic cancer cells (MDA‐MB‐468 and RKO) and normal cells (HEK‐293T). RKO cells (colorectal cancer) have been demonstrated as a reliable cell line for use in *CYPOR*‐mediated bioreductive experiments, hence their inclusion here as a positive control.^[^ ^51^ ^]^ Comparable *CYPOR* expression but significantly varied CD13 expression was observed across all tested cell lines (**Figure** **5**A,B). ICT10336 was incubated with RKO and MDA‐MB‐468 cells for 6 and 24 h in normoxic and hypoxic conditions. Bioreduction of ICT10336 was observed in both cells only in hypoxic conditions with the release of Leu‐AZD6738 and AZD6738 detected. However, the release of significant levels of free AZD6738 was observed only in MDA‐MB‐468 cells but not RKO cells, despite the high‐level release of Leu‐AZD6738 observed (Figure 5C – E). This observation corresponds to the observed lack of CD13 expression in RKO cells contrary to the high CD13 expression in MDA‐MB‐468 (Figure 2A,B), suggesting a role for CD13 activity in the release of free AZD6738 following bioreduction of ICT10336 as proposed. In addition, negligible levels of Leu‐AZD678 or AZD6738 were observed following ICT10336 incubation with normal cells (HEK293T), despite *CYPOR* expression (Figure 5F).

**Figure 6:**
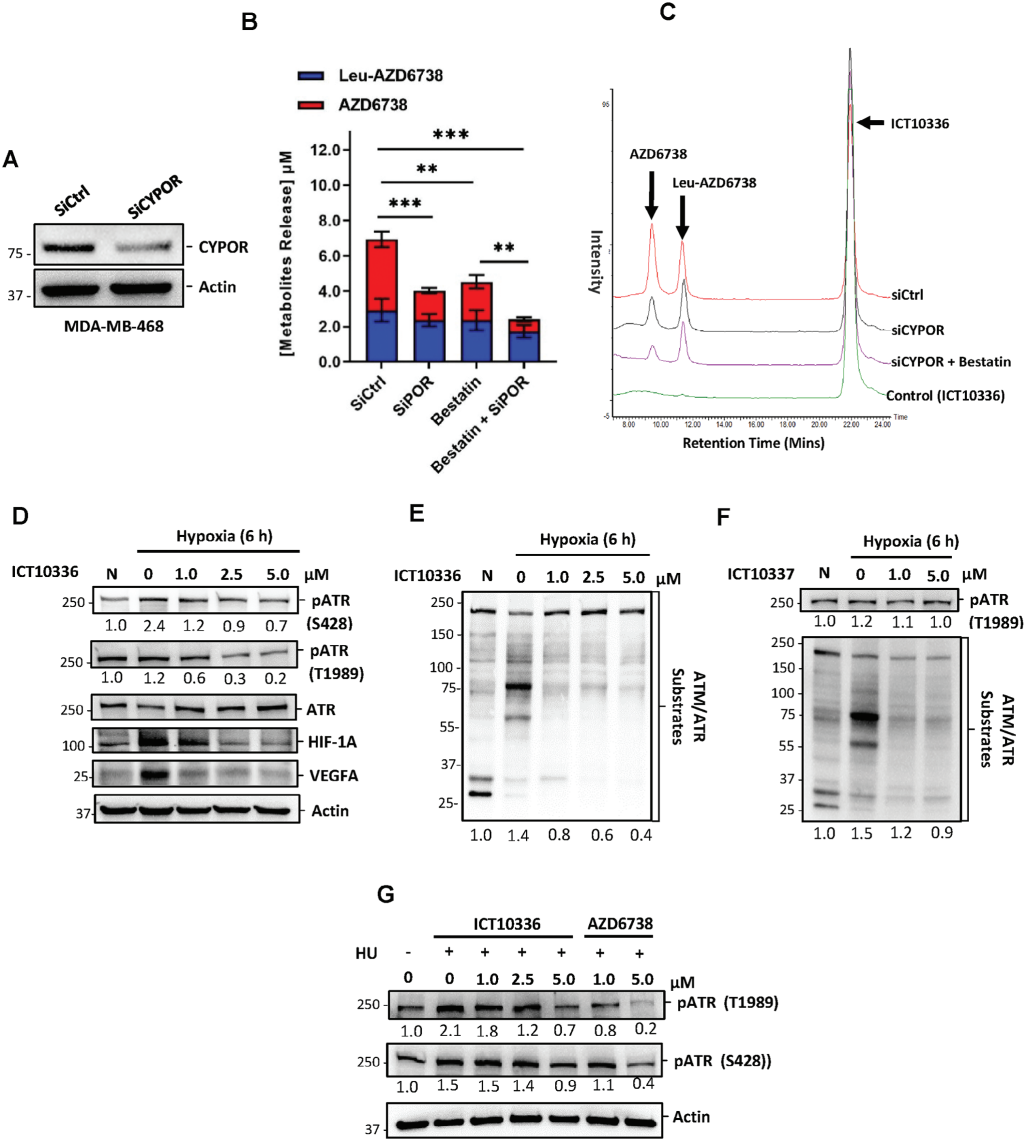
Cellular metabolism of ICT10336 abrogated hypoxia‐dependent ATR functions. A) Reduced expression of *CYPOR* protein in MDA‐MB‐468 cells following 48 h si*CYPOR* knockdown. B) Quantified in vitro cellular (combined intracellular and extracellular) concentrations of Leu‐AZD6738 and free AZD6738 following ICT10336 incubation with si*CYPOR* or bestatin treated hypoxic MDA‐MB‐468 cells. C) Representative of Selective Ion Recording (SIR) chromatogram demonstrating the role of *CYPOR* and CD13 in the hypoxic mediated metabolism of ICT10336. Western blot for markers of hypoxia‐mediated ATR activation in ICT10336‐treated D,E) and ICT10337‐treated F) in hypoxic MDA‐MB‐468 cell lysates. G) MDA‐MB‐468 cells were treated with Hydroxyurea(4.0 mm) for 6 h in the presence of AZD6738 or ICT10336. Cell lysates were probed for ATR activation using western blot.

Considering the presence of other reductases in cells other than *CYPOR*,^[^ ^50^, ^52^ ^]^ we further confirmed the specific contribution of *CYPOR* to the observed activation of ICT10336 and subsequent free AZD6738 release in hypoxic TNBC cells. To achieve this, we knocked down the expression level of *CYPOR* in MDA‐MB‐468 cells (**Figure** **6**A) and monitored the effect on the hypoxia‐mediated metabolism of ICT10336. Additionally, CD13 activity was inhibited using bestatin^[^ ^53^ ^]^ Both *CYPOR* expression knockdown and bestatin incubation significantly reduced ICT10336 activation and subsequent release of AZD6738 in hypoxic MDA‐MB‐468 cells (Figure 6B,C), demonstrating the role of *CYPOR* reductase and CD13 in the activation of ICT10336 as proposed. In addition, a 6‐hour treatment of hypoxic TNBC cells with ICT10336 was observed to significantly abrogate hypoxia‐dependent ATR phosphorylation and its subsequent functions including the phosphorylation of ATR/ATM substrates, HIF‐1A stabilization, and VEFGA expression (Figure 6D,E). This was not the case for negative control compound, ICT10337, although some minimal effects were observed at 5 µm (Figure 6F). To confirm that the observed ICT10336‐mediated ATR inhibition was hypoxia‐dependent, we elicited ATR phosphorylation in normoxia using hydroxyurea (HU)‐induced replicational stress and investigated the effect of ICT10336 on ATR phosphorylation in normoxic conditions. HU (4 mm) was incubated in the presence of ICT10336 or AZD6738 in MDA‐MB‐468 cells for 6 h. Compared to the observed inhibition of HU‐induced ATR phosphorylation with AZD6738, relatively little or no effect was observed with same concentration of ICT10336 (Figure 6G). This data demonstrates the hypoxia‐specific activation of ICT10336 and its subsequent inhibition of ATR‐mediated hypoxia adaptation in hypoxic TNBC cells.

### 2.7 Metabolic Stability and Ex Vivo Tumor Metabolism of ICT10336

**Figure 7:**
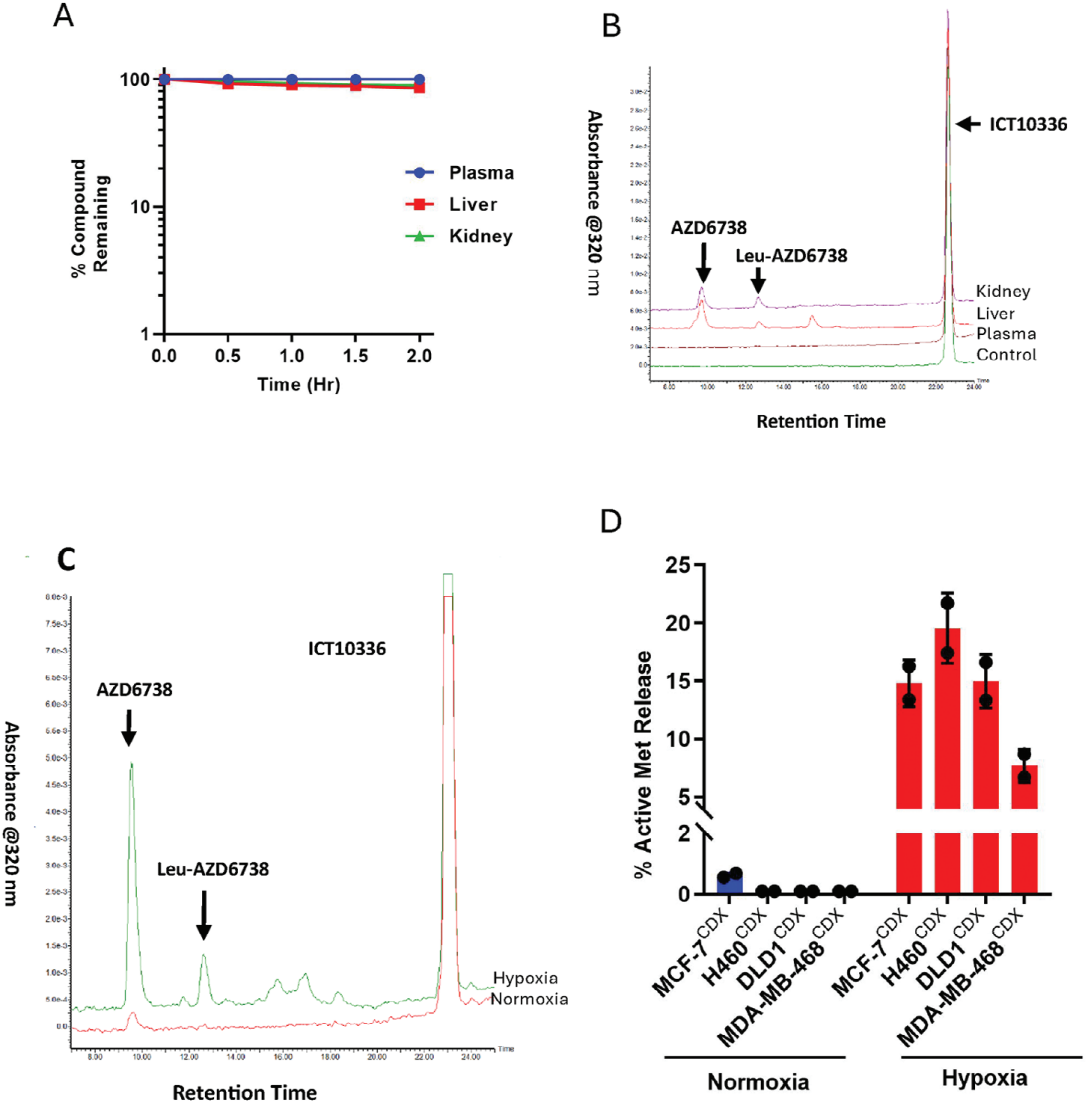
Metabolic stability of ICT10336 in normal mouse tissue homogenates, and selective activation in human cancer CDX in hypoxic conditions A) Quantified percentage of ICT10336 remaining in mouse liver and kidney homogenates and plasma during 2 h incubation in normoxic conditions; B) Representative LC‐MS chromatogram demonstrating the metabolic stability of ICT10336 in mouse normal tissues after 2 h of incubation; C) Representative LC‐MS chromatogram demonstrating differential metabolism of ICT10336 in normoxic and hypoxic MDA‐MB‐468 CDX homogenates after 1 h incubation. D) Quantified percentage release of active metabolites (combined Leu‐AZD6738 and free AZD6738) following ICT10336 incubation in normoxic and hypoxic human cancer CDX homogenates. Data shown are the mean of ≥ 2 independent experiments.

Metabolic stability of novel compounds remains critical to their efficacy and safety, in vivo. To predict the metabolic stability profile of ICT10336, particularly its susceptibility to *CYPOR* and protease activities in normal tissues, we assessed the metabolic vulnerabilities of ICT10336 in normal tissues using ex vivo models; mouse tissue (liver and kidney) homogenates and plasma. ICT10336 (10 µm) was incubated in normal tissue homogenates and plasma for 2 h at 37 °C, and its metabolism monitored by LC‐MS over time. ICT10336 demonstrated stability in all tested tissues with ≥ 92% compound remaining and ≤5% free AZD6738 release after 2 h incubation (**Figure** **7**A,B). This indicates the metabolic resistance of ICT10336 to normal tissue metabolism, thereby preventing unspecific release of AZD6738 in tissue. Next, we assessed the bioreduction possibility of ICT10336 and the release of AZD6738 in human cancer cell line‐derived xenograft (CDX) homogenates. This was performed by incubating ICT10336 (10 µm) in CDX homogenates, in both normoxic and hypoxic conditions for 1 h. Again, the release of active metabolite (Leu‐AZD6738 and free AZD6738) from ICT10336 was predominant (10 – 25%) only in hypoxic incubation as compared to ≤ 1.0% observed in normoxic conditions (Figure 7C,D; Figure S2, Supporting Information). In summary, this data emphasizes the hypoxia‐selective manner of AZD6738 release from ICT10336, and more importantly demonstrates the potential of ICT10336 in the in vivo setting.

### 2.8 Hypoxia‐Dependent Cytotoxicity of ICT10336 in TNBC Cells

**Figure 8:**
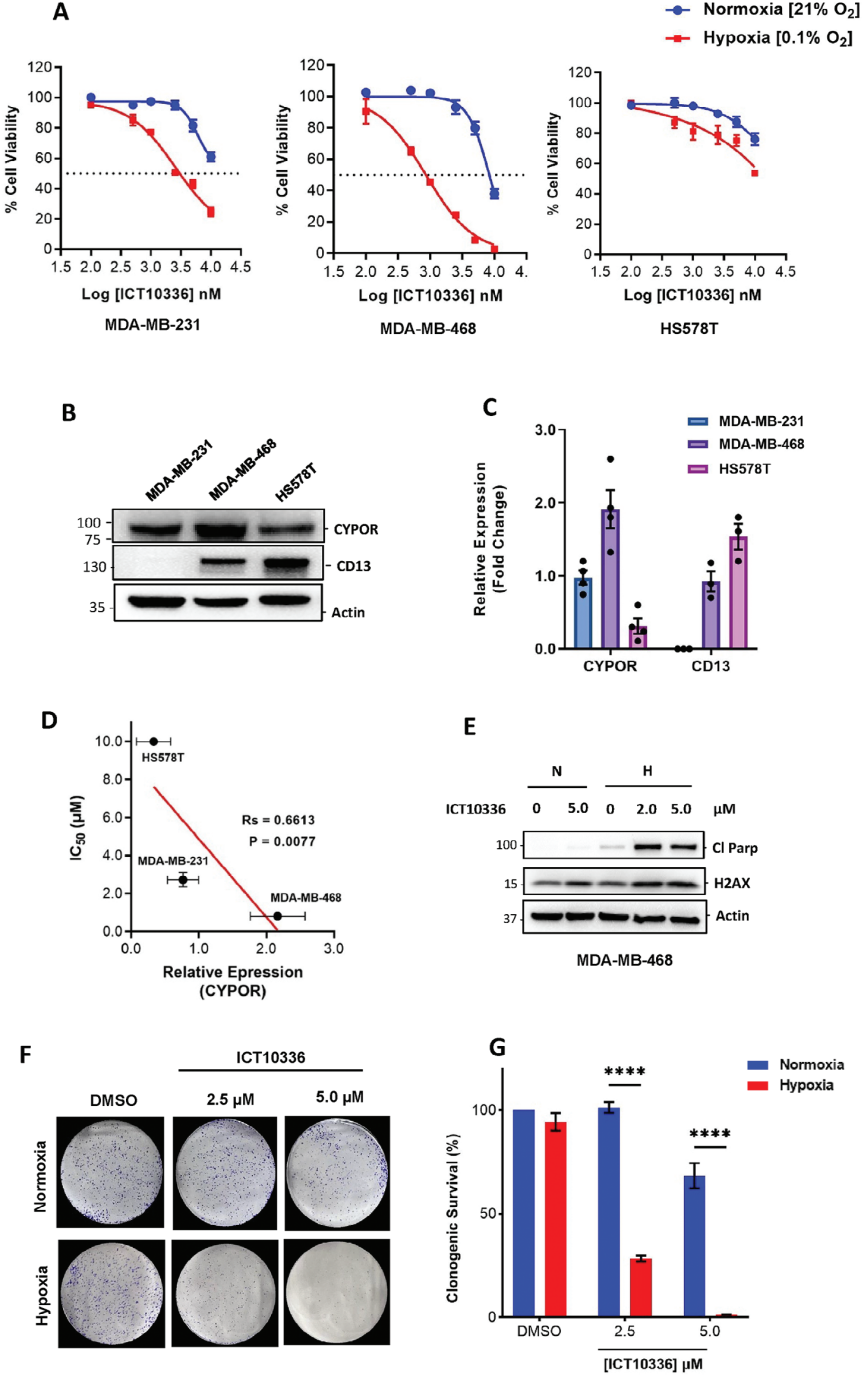
Hypoxia‐dependent cytotoxicity of ICT10336 in TNBC cells. A) Hypoxic TNBC cells were treated with various concentrations of ICT10336 in normoxic and hypoxic conditions for 24 h followed by 72 h regrowth in drug‐free medium in normoxic conditions. Cell viability was assessed via an MTT assay. *CYPOR* and CD13 protein expression in TNBC cell lines; B) Western blot using anti‐*CYPOR* and anti‐CD13 antibodies and C) quantified relative expressions of *CYPOR* and CD13 as detected. Band intensity was measured by Image Lab Software 6.1 and normalized to a β‐actin loading. D) Relationships between hypoxic cytotoxicity of ICT10336 (IC_50_) and *CYPOR* expression for tested TNBC cell lines. E) Lysates of MDA‐MB‐468 cells treated with ICT10336 for 6 h in normoxic and hypoxic conditions were analyzed for cleaved PARP and pH2AX expression. F,G) Clonogenic assay results after exposure of MDA‐MB‐468 cells to ICT10336 for 24 h in normoxic and hypoxic conditions and allowed to regrow in drug‐free medium in normoxic conditions for 12 days; Representative images of crystal violet stained colonies F), and relative quantification of the stained colonies in each treatment using ImageJ G). Data shown are the mean of ≥ 3 independent experiments ± SEM. ****p > 0.0001 (two‐way ANOVA).

As earlier demonstrated, ATR inhibition is critical for cell viability and proliferation in hypoxic TNBC cells (Figure 1). Hence, with the observed hypoxic‐dependent cellular AZD6738 release from ICT10336 and subsequent ATR inhibition, we evaluated the effect of ICT10336 on cell viability and proliferation of TNBC cells in normoxic and hypoxic conditions. TNBC cells were treated with ICT10336 for 24 h in either hypoxic or normoxic conditions, and allowed to regrow in drug‐free medium in normoxic conditions for 72 h. The cytotoxicity of ICT10336 in TNBC cells was observed to be hypoxia‐dependent, with HCR >4.0 in MDA‐MB‐231 cells and HCR ≈10 in MDA‐MB‐468 cells. However, no hypoxia‐dependent cytotoxicity of ICT10336 was observed in HS578T cells (**Figure** **8**A and **Table** **2**). With this said, 96 h continuous ICT10336 treatment of HS578T cells observed >3.6 HCR (Figure S3B, Supporting Information). To explain the varied hypoxia‐dependent cytotoxicity of ICT10336 observed in the tested TNBC cells, we assessed the expression of *CYPOR* and CD13 in these cells. As shown in Figure 8B,C, varied expression of *CYPOR* and CD13 was observed, with 6.2‐fold range in *CYPOR* expression across these cell lines. The *CYPOR* expression of these cell lines showed significant correlation with their relative ICT10336 sensitivity, IC_50_ (Figure 8D). This data emphasizes the role of *CYPOR* activities in the observed hypoxia‐mediated cytotoxicity of ICT10336 and explains the observed varied cytotoxicity. Again, 6 h treatment of hypoxic MDA‐MB‐468 cells with ICT10336 led to increased apoptosis (cleaved PARP) and increased DNA damage accumulation (p‐H2AX) as compared to negligible or little increase in normoxic control (Figure 8E). In addition, the colony‐forming ability of TNBC cells was also significantly inhibited in hypoxia ICT10336‐treated cells compared to normoxia‐treated controls (Figure 8F,G; Figure S3D, Supporting Information). Together this data demonstrates that ICT10336 is selectively active and cytotoxic in hypoxic cancer cells.

**Table 2:** IC_50_ values of ICT10336 in TNBC cells after 24 h treatment and 72 h regrowth in drug‐free medium in normoxic and hypoxic conditions.

TNBC	IC_50_ [µm]		HCR
	Normoxia	Hypoxia	
MDA‐MB‐231	>10	2.73 ± 0.38	**> 4.0**
MDA‐MB‐468	8.31 ± 0.46	0.81 ± 0.07	**10.3**
HS‐578T	>10	>10	–

### 2.9 ICT10336 is Less Toxic to Normal Human Healthy Cells than AZD6738

**Figure 9:**
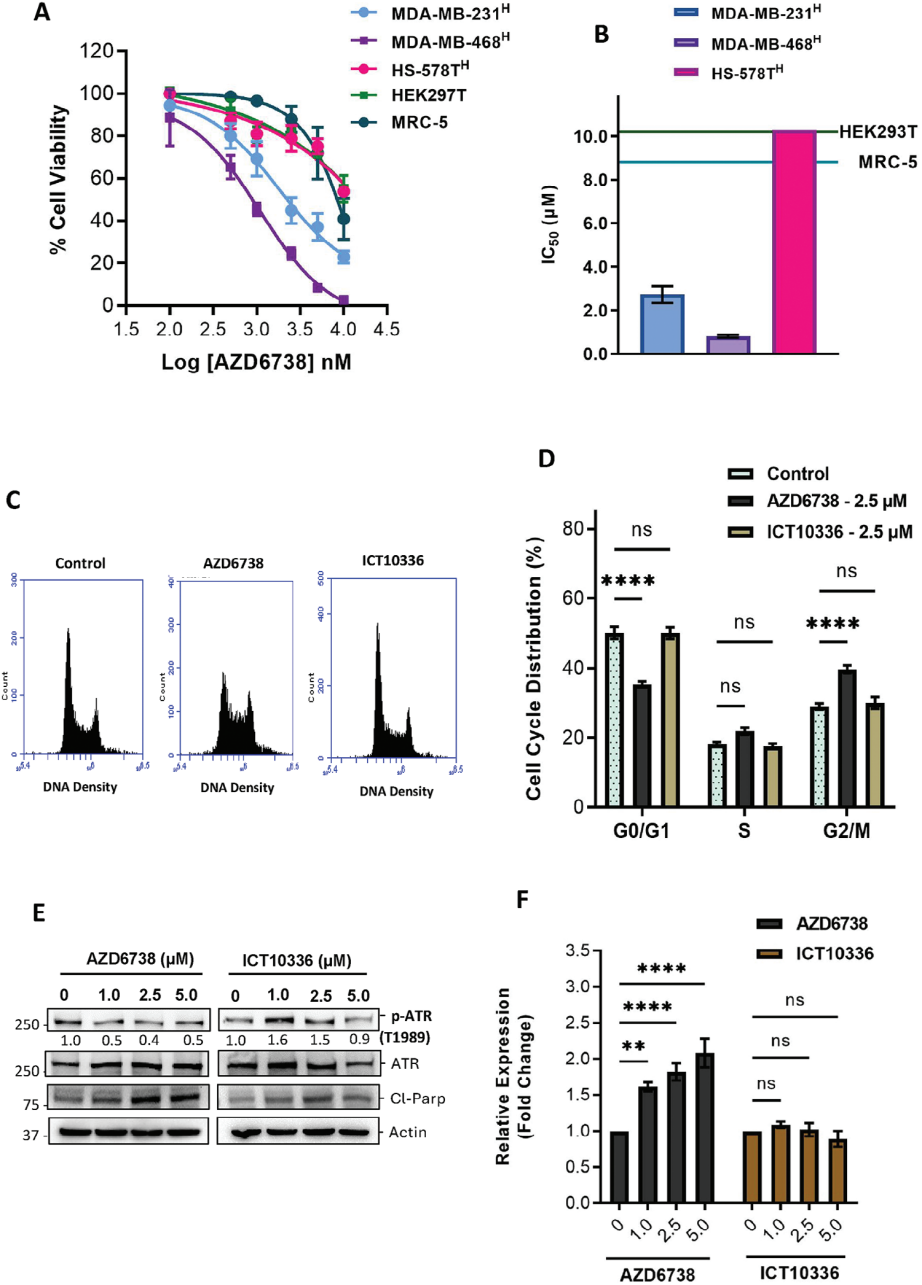
Prodrug, ICT10336, is less toxic in normal human cells compared to the active drug, AZD6738. A) Hypoxic TNBC cells and normoxic normal human cells were treated with various concentrations of AZD6738 for 24 h followed by 72 h regrowth in drug‐free medium in normoxic conditions. Cell viability was assessed via an MTT assay; B) Differential IC_50_ of ICT10336 in normal human cells and hypoxic TNBC cells; C,D) MRC‐5 cells treated with ICT10336 and AZD6738 were fixed with 66% ethanol and stained with propidium iodide. Cell cycle distributions of MRC‐5 cells were determined and quantified using flow cytometry. Normal human lung fibroblasts (MRC‐5) were treated with different concentrations of AZD6738 and ICT10336 for 24 h. Lysates were analysed for cleaved PARP and pH2AX expression; E) Western blot using p‐ATR (T1989), ATR, anti‐ cleaved PARP and pH2AX antibodies, and F) quantified relative expression of cleaved PARP as detected by anti‐cleaved PARP antibodies. Band intensity was measured by Image Lab Software 6.1 and normalized to a β‐actin loading. Data shown are the mean of ≥ 3 independent experiments ± SEM. *p > 0.05, **p > 0.01, ***p > 0.001 and ****p > 0.0001 (two‐way ANOVA).

Considering that the active drug (AZD6738) exerts dose‐dependent toxicity in normal tissues as shown (Figure 3) and previously reported,^[^ ^24^, ^26^ ^]^ we assessed the potential effects of ICT10336 on normal tissues. A chemosentitvity assay on normal human cell lines (MRC‐5 and HEK‐293T) with ICT10336 was conduted in normoxic conditions. ICT10336 was 3‐fold to <12‐fold less toxic in tested normal cells compared to the demonstrated chemosensensitivity in hypoxic TNBC cells (MDA‐MB‐231 and MDA‐MB‐468, **Figure** **9**A,B). In addition, compared to the sensitivity of tested normal cells to AZD6738, ICT10336 demonstrated 7‐fold to <12‐fold safety index in normal cells (Table S1, Supporting Information). ICT10336 at 3X IC_50_ concentration in hypoxic MDA‐MB‐468 cells demonstrated no significant effect on the cell cycle profile of normal lung fibroblasts (MRC‐5). Interestingly, AZD6738 at this same concentration induced G2/M arrest (Figure 9C,D). p‐ATR (T1989) is marker of active ATR,^[^ ^54^ ^]^ hence we investigated effect of ICT10336 on ATR activity in normal cells (MRC‐5 cells). ICT10336 demontrated no significant p‐ATR (T1989) inhibition compared to equimolar concentrations of AZD6738. However, increased levels of p‐ATR (T1989) were observed with ICT10336 treatment, paricularly at 1.0 and 2.5 µm. We further investigated the effect of ICT10336 on apoptosis in normal cells (Figure 9E,F). MRC‐5 cells incubated with ICT10336 (≈1X – 6X IC_50_ concentration in hypoxic MDA‐MB‐468) for 24 h showed little or no effect on both apoptosis and DNA damage accumulation. On the contrary, similar concentrations of AZD6738 on MRC‐5 cells was observed with significant increased levels of apoptosis (Figure 9E,F). This data demonstrates the potential of ICT10336 to selectively target hypoxic cancer cells whilst reducing toxicities on normal tissues

### 2.10 Hypoxia‐Selective Activity of ICT10336 in TNBC Spheroids

**Figure 10:**
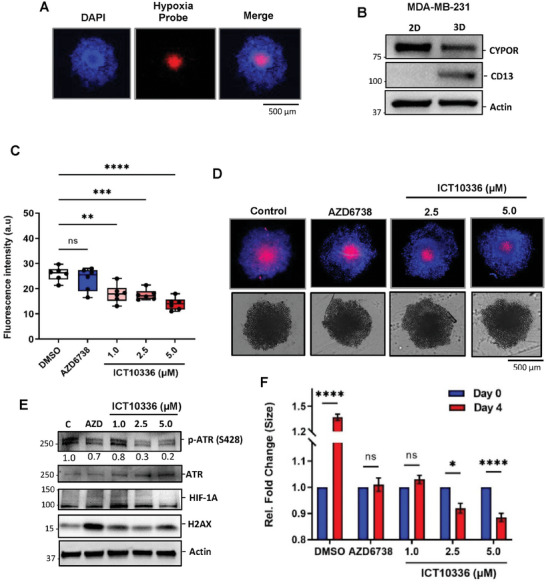
ICT10336 significantly reduces the hypoxic core in MDA‐MB‐231 spheroids. A) Confirmation of the presence of a hypoxic region within MDA‐MB‐231 spheroids. Scale bar 500 µm. MDA‐MB‐231 cells were grown as spheroids for 10 days and the presence of a hypoxic core was confirmed using the Image‐iT™ red hypoxia reagent. Hoechst dye was used as a nuclear marker; B) Western blot of spheroid lysates for *CYPOR* and CD13 expression (*n* = 2). Spheroids were treated with ICT10336 or AZD6738 for 4 days and further incubated with Image‐iT™ red hypoxia reagent; C) quantified fluorescence intensity using ImageJ (6 spheroids per condition); D) Representative images of MDA‐MB‐231 treated spheroids with and without incubation with Image‐iT™ red hypoxia reagent. Hoechst dye was used as a nuclear marker; E) Western blot of ICT10336 and AZD6738 treated MDA‐MB‐231spheroid lysates for markers of ATR‐mediated hypoxia adaptation mechanism; F) MDA‐MB‐231 spheroids were grown and treated with AZD6738 and ICT10336 for 4 days, and spheroid diameter measured with microscope reticle. 10 spheroids per condition. Data shown are the mean of 2 independent experiments ± SEM *p > 0.05, **p > 0.01, ***p > 0.001 and ****p > 0.0001 (two‐way ANOVA).

Poor drug penetration into hypoxic tumors remains a barrier to treatment and contributes to therapeutic resistance.^[^ ^40^ ^]^ Hence, using a 3D spheroid model, we tested the potential of ICT10336 to penetrate across a multicellular spheroid into the hypoxic core. First, MDA‐MB‐231 cells were grown as spheroids for 10 days, and the presence of a hypoxic core confirmed using Image‐iT hypoxia probe (**Figure** **10**A). The Image‐iT hypoxia probe is a live cell penetrant fluorogenic compound which only fluoresces in low oxygen environments^[^ ^55^ ^]^ We further assessed the expression of *CYPOR* and CD13 in these 3D spheroids and observed reduced *CYPOR* but increased CD13 expression compared to 2D controls (Figure 10B). It is worth stating that CD13 expression in MDA‐MB‐231 spheroids was only detected after deglycosylation of protein lysates. This observation is consistent to our recent report which demonstrated the existence cancer‐specific CD13 glycoforms in human breast CDX.^[^ ^56^ ^]^ Interestingly, treatment of spheroids with ICT10336 for 4 days (at non‐toxic concentrations to normal cells, Table S1, Supporting Information) significantly diminished the fluorogenic response of the hypoxia probe compared to DMSO control and AZD6738‐treated spheroids (Figure 10C,D). This reduced fluorogenic response of the hypoxia probe indicates a reduced hypoxic region within the ICT10336‐treated spheroids, which suggests the ability ICT10336 to efficiently reach and be subsequently bioreduced in the hypoxic core of MDA‐MB‐231 spheroids. We further confirmed this observation by probing treated spheroids for hypoxia‐dependent ATR activation and subsequent HIF‐1A mediated adaptation as earlier demonstrated in 2D models. Activation of ATR (S428 phosphorylation) and HIF‐1A protein stabilization was observed in MDA‐MB‐231 spheroids. However, this was inhibited only in the presence ICT10336 but not with AZD6738. This demonstrates the superior multicellular penetration of ICT10336, and subsequent activity compared to AZD6738. In addition, an increase level of H2AX phosphorylation was observed with ICT10336, particularly at 5 µm, indicating DNA damage accumulation effects possibly on neighboring non‐hypoxic proliferating cells. However, this effect (H2AX phosphorylation) was extremely high with AZD6738 treatment as expected with ATR inhibition on proliferating cancer cells (Figure 10E). In addition, significant reduction in spheroid size was observed only when ICT10336 treated, though at 2.5 and 5.0 µm. Taken together this data demonstrates the selective activation of ICT10336 in tumor hypoxia and subsequent activity on tumor hypoxic cells with potential a bystander effect on neighboring non‐hypoxic proliferating cells.

## 3 Discussion

ATR kinase is a major DNA damage repair protein whose inhibitors have emerged as a promising strategy for cancer treatment and are currently being evaluated in pateints with various solid cancers.^[^ ^21^, ^57^ ^]^ However, recent evidence suggests the possibility of non‐DDR functions of ATR in tumor hypoxia, which are critical for the survival of hypoxic cancer cells.^[^ ^32^, ^33^ ^]^ Consistent with these reports, we demonstrated that hypoxia sensitised TNBC cells to AZD6738, an ATR inhibitor (Figure 1), through the inhibition of HIF‐1A mediated hypoxia reponses (Figure 2). Tumor hypoxia is a unique feature of most solid tumors including treatment‐resistant TNBCs and remains a barrier to their successful treatment.^[^ ^42^, ^58^ ^]^ Thus, in this study, we demonstrate the potential of hypoxia‐specific delivery of AZD6738 to selectively eradicate hypoxic cancer cells, whilst minimising the normal cell toxicities associated with ATR inhibition. ATR is an essential protein whose inhibition is lethal to normal cells^[^ ^19^, ^48^ ^]^ as we have shown in normal human embryonic kidney cells and human lung fibroblasts with AZD6738 (Figure 3). It is therefore of no surprise that systemic toxicities remain a current challenge to the clinical potential of ATR inhibitors.^[^ ^26^, ^57^ ^]^

We have described a newly developed hypoxia‐activated prodrug (HAP) of AZD6738, ICT10336, which facilitates its delivery to the hypoxic microenvironment, where it is selectively activated to release active AZD6738, thus selectively inhibting hypoxia‐activation of ATR (Figure 4). Importantly, ICT10336 exerts little to no effect on ATR function in normoxia with substantial metabolic stability in normal tissues, and is less toxic to normal cells compared to AZD6738 (Figure 6). The identification of the oxidoreductases responsible for hypoxia‐dependent activation of bioreductive prodrugs remains critical for their rational clinical development.^[^ ^59^ ^]^ We have demonstrated that ICT10336 is a metabolic substrate of *CYPOR* activity and that the expression level of *CYPOR* in hypoxic TNBC cells correlates to AZD6738 release from ICT10336 and its subsequent activity (Figures 4 and 5). In addition to this, we further demonstrated that CD13 activity is a key complement to *CYPOR*‐mediated metabolism of ICT10336. Unlike the design of most reported HAPs which directly conjugate a bioreductive motif to the compound of interest,^[^ ^43^ ^]^ the design of ICT10336 incoporates the CD13 substrate, Leucine, inbetween the bioreductive motif and AZD6738. CD13 is a moonlighting protein with established roles in hypoxia‐related processes including angiogenesis .^[^ ^45^, ^60^ ^]^ It is worth stating that the inclusion of the CD13 substrate in the design of the produg was to facililate the readily release of free AZD6738 subsequent to hypoxia activation as this was observed to be compromised with direct conjugation. However, considering the high peptidase activty of CD13^[^ ^61^ ^]^ and its tumor‐specific isoform expression in breast cancers,^[^ ^56^ ^]^ this inclusion provides additional tumor selectivity.

Hypoxia activation of major DDR proteins, particularly ATR and DNA‐PK, has recently been reported in various cancers, including breast cancers.^[^ ^32^, ^62^, ^63^ ^]^ These observations perhaps explains to the failure of most reported HAPs, which predominantly focused on hypoxia release of cytoxic DNA damaging agents,^[^ ^41^, ^64^ ^]^ considering that DDR mechanisms in tumor hypoxia are now known to be already activated prior treatment. Activation of the DDR pathways prior to treatment has been shown to increase in the treatment‐induced DNA damage repair capacity of cancer cells and contributes to treatment resistance.^[^ ^66^, ^67^ ^]^ This hypothesis is consistent with recent report which demonstrated that the anti‐tumor activity of TH‐302 (a hypoxia activated cytotoxic agent) is greatly enhanced by the inhibtion of the ATR‐CHK1 pathway.^[^ ^65^ ^]^ Thus, hypoxia‐selective delivery of ATR inhibitors, as we have demonstrated with ICT10336, may not only improve the therapeutic window of ATR inhibitors but it may also potentiate DNA‐damaging therapies in tumor hypoxia. We have demonstrated a superior multicelluar penetrating ability of ICT10336 (cLogP: 2.47) to diffuse into the hypoxic core of 3D spheriods compared to AZD6738 (cLogP: 0.59) (Figure 10). This supports future combination strategies of ICT10336 with chemo‐ and radiotherapies in other highly hypoxic tumors beside TNBCs, particularly pancreatic,^[^ ^68^ ^]^ ovarian,^[^ ^69^ ^]^ and glioblastoma^[^ ^70^ ^]^ tumors. ATR activation has recently been demonstrated as a prognostic biomarker in recurrent ovarian cancer, a highly hypoxic cancer with ATR inhibition shown to be a promising therapeutic approach.^[^ ^37^ ^]^ PARP inhibitors are an approved treatment for TNBC patients.^[^ ^71^ ^]^ However, hypoxia in TNBCs has been shown to reduce the efficacy of PARP inhibition, with selective eradication of hypoxic breast cancer cells shown to substantially improving PARPi efficacy.^[^ ^72^ ^]^ Thus, this provides an opportunity for the combination of PARP inhibition and ICT10336 in TNBCs treatment.

## 4 Conclusion

In conclusion, we have developed a new, non‐toxic HAP of AZD6738 that selectively releases free AZD6738 in the hypoxic microenvironment thereby selectively targeting hypoxia‐elicted functions of ATR, which abrogates the survivial mechanisms of hypoxic TNBC cells in both 2D cell culture and 3D spheriod models. This approach demonstrates a superior therapeutic strategy for an ATR inhibitor to selectively target treatment‐resistant hypoxic cancer cells whilst providing local bystander effect on neighbouring non‐hypoxic TNBC cells, and in addition provide a means of reducing systemic toxicities associated with ATR inhibitors. Thus, our data supports further development of this concept for further clinical applications of ATR inhibitors.

## 5 Experimental Section

### Materials

Bactosomal human NADPH‐CYP reductase (CYP004, Cypex); Cleaved parp (#9541), p‐ATR (S428) (#2853), ATR (#2790), β‐actin (#3700), p‐H2AX (S139) (#2577) antibodies were purchased from Cell Signalling Technology, UK. anti‐GLUT‐1 (#66290‐1‐lg) and anti‐VEGFA (#19003‐1‐AP) from Proteintech, UK. HIF‐1A antibody (#610 958) from BD Bioscience, BELG. p‐ATR (T1989) (GTX128145) antibody from Genetex, UK. CYPOR antibody form Santa Cruz Bestatin, Crystal violet, DMEM media, Fetal bovine serum (FBS) and phosphate buffered saline (PBS) from Merck, UK. HRP–conjugated anti‐rabbit and anti‐mouse secondary antibodies were obtained from DAKO, UK. Image‐IT hypoxia reagents and SiCYPOR from ThermoFisher, UK. All other chemicals were of analytical grade.

### Cell Culture

Human colorectal carcinoma cell line; RKO, human breast adenocarcinoma cell lines; MDA‐MB‐231, MDA‐MB‐468, and Hs578T, and human non‐cancer cell line; HEK293T, and MRC‐5, were obtained from American Type Culture Collection (ATCC). Cells were cultured in DMEM media supplemented with 10% (v/v) foetal bovine serum, sodium pyruvate (1 mm), and l‐glutamine (2 mm) in a humidified incubator at 37 °C with 5% of carbon dioxide. Cell lines were used at low passage (<12 passages) for less than 6 months. Hypoxic condition (0.1% O_2_) was achieved using Whitley H35 Hypoxystation (Don Whitley Scientific, UK)

### Western Blotting

Cell pellets were lysed by resuspension in Pierce IP lysis buffer with protease and phosphatase inhibitors, and incubated on ice for 30 min, sonicated and centrifuged (10,000 g, 10 min, 4 °C). Supernatants were pipetted, and total protein concentrations determined using a BCA protein assay kit (Thermo Fisher Scientific, UK). Protein (40 µg) of total cell lysate was separated by 4–15% Mini‐PROTEAN TGX Gels (Bio‐Rad, UK) and then transferred onto nitrocellulose membranes. Membranes were blocked with 5% dry milk or 5% BSA in PBST. Membranes were probed for specific protein expression with respective antibodies, followed by horseradish peroxidase (HRP)‐conjugated secondary antibodies. These immunoblots were visualized and analyzed using ChemiDoc Imaging System with Image Lab Software 6.1.

### Bactosomal Human NADPH‐CYP Reductase Assay

Bactosomal human NADPH‐CYP reductase (100 pmol mL^−1^) was added to an Eppendorf tube containing buffer (100 mm KPi pH 7.4), MgCl_2_ (5 mm), and compounds (10 µm) under normoxic (21% O_2_) or hypoxic (0.1% O_2_) conditions. NADPH (1 mm) was added to the incubating mixture and reaction aliquots (100 µL) taken at different times, into eppendorf tubes containing DCM (200 µL). Tubes were centrifuged (4500 g, 2 min) with 200 µL of the bottom organic layer carefully removed into separate tubes, and dried using SP Genevac EZ‐PLUS evaporator for 30 min. The dried reaction was dissolved in 50 µL of 90% MeOH, 10% H_2_O, 0.1% formic acid and transferred into HPLC vial for LC‐MS analysis.

### In Vitro Metabolism

Cellular activation and metabolism of ICT10336 in normoxic or hypoxic conditions was conducted as described previously^[^ ^46^ ^]^ with slight modifications. Cells (2 × 10^6^/mL) in complete media were seeded into 6‐well (1 per time point plus controls), and allowed attached over‐night. Pre‐conditioned media (2 mL) containing ICT10336 (10.0 µm) were added to each PBS washed cells. Controls contained either no cells or no drug. Samples were incubated for 6 and 24 h at 37 °C in either normoxia or hypoxic conditions. At each indicated time point, medium was removed and stored at −80 °C. Cells were washed (twice) with ice‐cold PBS, stripped and centrifuged (1000 g, 2 min), and the resulting cell pellets were solubilized in acetonitrile (200 µL), and then sonicated to disrupt the cells. Samples were centrifuged (10 000 g, 10 min), with the supernatant collected. Supernatants were then dried using a SP Genevac EZ‐PLUS evaporator for 30 min. The dried reaction was dissolved in a solution of 90% MeOH, 10% H_2_O, 0.1% formic acid (50 µL), which was then transferred into an HPLC vial for LC‐MS analysis.

### Ex Vivo Metabolism

Homogenized (1:4, w/v) human cancer cell‐derived tumors xenografts (CDX), normal mice tissues (liver and kidney, and plasma) were incubated with IC10336 (10 µm) in either hypoxic or normoxic conditions with reaction aliquots removed over a time‐period. Proteins were precipitated using acetonitrile and centrifuged for supernatant. Supernatants were dried for 30 min in a SP Genevac EZ‐PLUS evaporator. Dried metabolites were dissolved in 90% MeOH, 10% H_2_O, 0.1% formic acid (50 µL), and analyzed on LC‐MS.

### LC‐MS Analysis

Stock solutions of AZD6738, Leu‐AZD6738 and ICT10336 were prepared at 10 mm in DMSO and stored at −20 °C. Standard solutions for system calibration were made by further dilutions of stock solutions with a mixture of 10% mobile phase A (MPA) and 90% mobile phase B (MPB). For each set of experiments run on LC‐MS, calibration curves were created between 0.1 and 2 µm. LC‐MS of samples was carried out using a gradient method (**Table** **3**) described below using a HiChrom RPB column (25 cm x 2.1 mm id; HIRPB‐250AM; R6125) on a Waters Alliance 2695 HPLC (Micromass, Manchester, UK) with a photodiode array detector, connected in series with a Waters Micromass ZQ quadrupole mass spectrometer in ESI^+^ mode. [MS ESI+ source parameters: Desolvation gas; 650 l/h, cone gas; 50 l/h, capillary voltage; 3 kV, extraction voltage; 5 V, cone voltage; 20 V, Rf voltage; 0.2 V, source block temperature; 120 °C and desolvation temperature; 350 °C]. ICT10336 and its respective metabolites, including AZD6738, Leu‐AZD6738 were analyzed using UV absorbance at 320 nm, and set single ion recordings. Samples were maintained at 4 °C in the auto‐sampler and 10 µL was injected for analysis. To determine the cellular drug concentrations of ICT10336 and its metabolism, the following equation was applied (all volumes in µl): Dilution factor = [(volume of a single cell x 1 × 10^6^ cells) + 50 µl] / volume of a single cell x 2 × 10^6^ cells. Raw LC‐MS data were processed using Mass Lynx V4.1. Graphs were plotted using Graph Pad Prism 8.

**Table 3:** Gradient method.

Time (min)	MPA (%)	MPB (%)
0	60	40
15	40	60
25	0	100
26	60	40

Run Time = 35 min; Injection Vol = 10 µl; MPA = 90% Water, 10% Methanol; MPB = 10% Water, 90% Methanol;

### Quantitative RT‐PCR

VEGFA Gene expression was determined by quantitative reverse transcription‐PCR. RNA was extracted from cells after treatment using RNeasy Micro Kit (Qiagen). Complementary DNAs (cDNAs) were produced using High‐Capacity cDNA Reverse Transcription Kit (ThermoFisher, UK). qPCR was performed using SYBR Green PCR Master Mix (Primer Design, UK). The B2M gene was used as an endogenous control. The β‐actin gene was used as an endogenous control. VEGFA primer (HP100106) and B2M primer (5′‐CCAAGGAAGGCGTCTAAGGC‐3′ and 5′‐CTTTCGAGCGCAACCACTTTG‐3′) were used.

### Cytotoxicity Assay

In vitro chemosensitivity of cells to compounds was determined using the 3‐(4,5‐dimethylthiazol‐2‐yl)−2,5‐diphenyltetrazolium bromide (MTT) assay. Cells (1000–3000/well) were seeded in 96‐well plates and incubated overnight at 37 °C with 5% CO_2._ Cells were treated with compound or solvent (DMSO) for 96 h in normoxic (21% O_2_) or hypoxic conditions (0.1% O_2_) conditions or 24 h either in normoxic (21 O_2_%) or hypoxic conditions (0.1% O_2_) conditions after which medium was replaced with drug‐free medium for 72 h in in normoxic (21% O_2_). DMSO concentrations did not exceed 0.1%, which was not toxic at this concentration. After compound treatment, the chemosensitivity of the cells was assessed, and cell survival post‐treatment determined. Survival curves were obtained and IC_50_ values calculated using GraphPad Prism 8.

### Clonogenic Assay

Cells (500 cells per well) in 6‐well plate were treated with compounds or solvent (DMSO) for 24 h in normoxic (21% O_2_) or hypoxic conditions (0.1% O_2_) conditions after which medium was replaced with drug‐free medium. Visible colonies were left to form for 10 days and stained with 0.5% crystal violet (dissolved in 25% methanol in PBS). Colonies were counted using ImageJ software.

### Cell Cycle Analysis

MRC‐5 cells were seeded at a density of 1 × 10^6^ cells and incubated for 24 h. Cells were treated with either AZD6738 or ICT10336 for 24 h. Treated cells and controls were trypsinised, washed with PBS, and fixed with 66.6% ethanol overnight. The fixed cells were rinsed with PBS, labeled with FxCycle PI/RNase staining solution following the manufacturer's instructions (Thermo‐Fisher Scientific, UK), and analyzed by flow cytometry (BD Accuri C6 Plus Flow Cytometer).

### Multicellular Tumor Spheroids

Spheroids were formed using the aggregation method, MDA‐MB‐231 (2000 cells) were seeded per well into Biofloat‐Flex‐coated 96‐well plate (faCellitate, UK) and centrifuged at 500 g for 5 min. Cells were allowed to aggregate forming a single mass of cells under standard cell culture conditions (37 °C, 5% CO_2_) for 10 days. The presence of hypoxic core was confirmed using Image‐iT Red Hypoxia Reagent (ThermoFisher, UK). Spheroids, 10 per treatment condition were then treated with compounds for 4 days.

### Quantification and Statistical Analysis

All results were expressed as mean ± standard error of measurement (SEM) from at least 3 independent experiments. The statistical analysis was performed using GraphPad Prism 8 (GraphPad Software, San Diego, CA, USA). *P* < 0.05 was considered statistically significant.

### General Information on Compund Synthesis

NMR spectra were recorded on a Bruker AMX 400 NMR spectrometer and are reported in parts per million (ppm) on the δ scale relative to residual CDCl3 (δ 7.26 or δ 77.0). The progress of reactions was monitored by analytical thin layer chromatography (Merck, TLC 60 F254 plates) and/or by LC/MS using Waters Alliance 2695 Separations Module, Waters 996 PDA Detector, Waters Micromass ZQ Mass Detector. TLC plates were visualized first with UV (254 nm). High‐resolution mass spectra were recorded using Thermo scientific, LTQ Orbitrap no. 01289B. Column chromatography was performed using silica gel (230–400 mesh). The solvent compositions for all separations were on a volume/volume (v/v) basis. All solvents were of reagent grade. Chemicals and solvents were purchased from Fluorochem, Fischer, Fmoc‐ Novabiochem. AZD6738 was purchased from Euroasias.

### Synthesis of 3*R*)−3‐methyl‐4‐(6‐(1‐(*S*‐methylsulfonimidoyl‐(*N*‐(4‐nitrobenzylcarbamoyl))cyclopropyl)−2‐(1*H*‐pyrrolo[2,3‐*b*]pyridin‐4‐yl)pyrimidin‐4‐yl)morpholine (NBC‐AZD6738) (4)

*p*‐Nitrobenzyl chloroformate (15.2 mg, 71 µmol) and pyridine (9.5 µL, 118 µmol) were added to a solution of AZD6738 (24 mg, 59 µmol) in anhydrous CH_2_Cl_2_ (1.2 mL) at 0 °C and the reaction was allowed to warm to room temperature (RT). After 15 min, the volatiles were removed under reduced pressure, and the residue was purified by column chromatography on silica gel (CH_2_Cl_2_:MeOH 95:5) to afford a mixture of regioisomers. Further purification of this mixture by column chromatography on silica gel (Hexane/EtOAC/MeOH 60:40:4) afforded compound 4 (18 mg, 51%) as a pale yellow solid – ^1^H NMR (CDCl_3_, 400 MHz) δ 10.03 (bd, 1H, NH), 8.42 (d, 1H, *J*_3_ = 5.0 Hz), 8.15 (d, 2H, *J*_3_ = 8.8 Hz), 8.04 (d, 1H, *J*_3_ = 5.0 Hz), 7.47 (m, 1H), 7.45 (d, 2H, *J*_3_ = 8.8 Hz), 7.31 (dd, 1H, *J*_H,NH_ = 2.0 Hz, *J*_3_ = 3.4 Hz), 6.81 (s, 1H), 5.19 (d, 1H, *J*_2_ = 13.4 Hz), 5.11 (d, 1H, *J*_2_ = 13.4 Hz), 4.42 (m, 1H), 4.22 (d, 1H, *J* = 14.4 Hz), 4.06 (dd, 1H, *J* = 3.6 Hz, *J* = 11.6 Hz), 3.84 (d, 1H, *J* = 11.6 Hz), 3.73 (dd, 1H, *J* = 3.2 Hz, *J* = 11.6 Hz), 3.56 (ddd, 1H, *J* = 2.8 Hz, *J* = 11.6 Hz, *J* = 14.4 Hz), 3.48 (s, 3H), 3.35 (ddd, 1H, J = 4.0 Hz, *J* = 11.6 Hz, *J* = 12.9 Hz), 2.26 (m, 1H), 1.89 (m, 1H), 1.73 (m, 1H), 1.61 (m, 1H), 1.34 (d, 3H, *J* = 6.8 Hz); ^13^C NMR (CDCl_3_, 100 MHz) δ 163.95, 162.57, 160.88, 158.57, 150.30, 147.69, 143.78, 142.72, 137.75, 128.21 (2C), 126.39, 123.82 (2C), 116.81, 115.63, 103.44, 102.87, 70.98, 66.81, 66.25, 47.61, 46.37, 40.28, 39.53, 14.25, 13.83, 12.81; HMRS (ESI+) Found 592.19599 calculated. for C_28_H_30_N_7_O_6_S 592.19783 [M+H]^+^.

### Synthesis of (2S)−2‐amino‐4‐methyl‐N‐(methyl(1‐(6‐((R)−3‐methylmorpholino)−2‐(1H‐pyrrolo[2,3‐b] pyridin‐4‐yl)pyrimidin‐4‐yl)cyclopropyl)(oxo)‐l6‐sulfaneylidene)pentanamide. (Leu‐AZD6738) (5)

Fmoc‐Leu‐OH (129 mg, 0.363 mmol 1.5 eq.) was dissolved in dry CH_2_Cl_2_ (6 µL). Diisopropylcarbodiimide (76 µL, 0.484 mmol, 2.0 eq.) was added and continually stirred for 15 min. AZD6738 (100 mg, 0.242 mmol, 1.0 eq.) was then added and stirred for a minimum of 48 h. Crude synthesised compound was purified by column chromatography on silica gel [petroleum ether (PET): EtOAc (3:2); 4% MeOH] to obtained compound **5** (73%) as a white solid. ^1^H NMR (400 MHz, CDCl_3_) δ 10.22 (bs, 1H, NH), 8.41 (d, 1H, *J*_3_ = 5.2 Hz), 8.01 (d, 1H, *J*_3_ = 5.2 Hz), 7.47 (d, 1H, *J*_3_ = 3.6 Hz), 7.30 (d, 1H, *J*_3_ = 3.6 Hz), 6.74 (s, 1H), 4.55 (bs, 1H), 4.12 (m, 1H), 4.06 (dd, 1H, *J* = 4.0 Hz, *J* = 11.2 Hz), 3.85 (d, 1H, *J* = 11.6 Hz), 3.74 (dd, 1H, *J* = 2.8 Hz, *J* = 11.6 Hz), 3.60 (ddd, 1H, *J* = 2.8 Hz, *J* = 11.6 Hz, *J* = 12.4 Hz), 3.50 (s, 3H), 3.39 (dd, 1H, *J* = 4.0 Hz, *J* = 12.4 Hz), 3.33 (dd, 1H, *J* = 5.2 Hz, *J* = 9.2 Hz), 2.49 (bs, 2H, NH_2_), 2.21 (m, 1H), 1.88 (m, 1H), 1.72 (m, 1H), 1.65 (m, 1H), 1.53 (m, 1H), 1.39 (d, 3H, *J* = 6.8 Hz), 1.12‐17 (m, 2H), 0.84 (d, 3H, *J* = 6.4 Hz), 0.811 (d, 3H, *J* = 6.4 Hz); ^13^C NMR (100 MHz, CDCl_3_) δ 184.81, 163.73, 162.31, 160.96, 150.17, 142.56, 137.75, 126.31, 118.64, 115.58, 103.20, 102.67, 70.96, 66.70, 56.17, 47.28, 46.51, 44.30, 40.36, 39.50, 24.89, 23.21, 22.63, 22.63, 13.93, 12.97; HRMS (ESI+) Found 526.2588 calculated for C_26_H_36_N_7_O_3_S 526.2595 [M+H]^+^.

### Synthesis of (*S*)−4‐Nitrobenzyl {4‐methyl‐1‐[(3′*R*)−3′‐methyl‐4′‐(6′‐(1′‐(*S*‐methylsulfonimidoyl)cyclo propyl)−2′‐(1*H*‐pyrrolo[2,3‐*b*]pyridin‐4′‐yl)pyrimidin‐4′‐yl)morpholine]−1‐oxopentan‐2‐yl}carbamate (Nitrobenzyl carbamate‐Leu‐AZD6738, ICT10335) (6)

Leu‐AZD6738 (20 mg, 38 µmol) and DMAP (22 mg, 180 µmol) were added to a solution of **1** (24 mg, 76 µmol) in anhydrous CH_2_Cl_2_ (2.2 mL), and the reaction was stirred at RT overnight. Afterward, the volatiles were removed under reduced pressure, and the residue was purified by column chromatography on silica gel (CH_2_Cl_2_:MeOH 99:1→97:3→95:5→90:10) to afford compound **6** (21 mg, 79%) as a pale yellow solid. ^1^H NMR (400 MHz, CDCl_3_) δ 9.86 (s, 1H, NH), 8.34 (d, 1H, *J*_3_ = 4.8 Hz), 8.02 (d, 2H, *J*_3_ = 8.6 Hz), 7.94 (d, 1H, *J*_3_ = 4.8 Hz), 7.39 (dd, 1H, *J*_H,NH_ = 2.4 Hz, *J*_3_ = 3.2 Hz), 7.27 (d, 2H, *J*_3_ = 8.6 Hz), 7.22 (dd, 1H, *J*_H,NH_ = 1.6 Hz, *J*_3_ = 3.2 Hz), 6.67 (s, 1H), 5.39 (d, 1H, *J* = 8.8 Hz), 5.02 (d, 1H, *J*_2_ = 13.6 Hz), 4.98 (d, 1H, *J*_2_ = 13.6 Hz), 4.48 (m, 1H), 4.13 (m, 1H), 3.98 (dd, 1H, *J* = 3.6 Hz, *J* = 11.6 Hz), 3.78 (d, 1H, *J* = 11.6 Hz), 3.67 (dd, 1H, *J* = 2.8 Hz, *J* = 11.6 Hz), 3.55 (ddd, 1H, *J* = 2.8 Hz, *J* = 11.6 Hz, *J* = 12.4 Hz), 3.46 (s, 3H), 3.30 (ddd, 1H, *J* = 3.6 Hz, *J* = 11.6 Hz, *J* = 12.4 Hz), 2.22 (m, 1H), 1.90 (m, 1H), 1.76 (m, 1H), 1.60‐1.54 (m, 2H), 1.52 (m, 1H), 1.41 (d, 1H, 3H, *J* = 6.8 Hz), 1.26 (m, 2H), 0.89 (d, 6H, *J* = 4.0 Hz); ^13^C NMR (100 MHz, CDCl_3_) δ 180.00, 162.71, 161.31, 159.52, 154.38, 149.17, 146.40, 142.98, 141.74, 136.61, 126.73 (2C), 125.15, 122.57 (2C), 117.46, 114.57, 102.15, 101.69, 69.89, 65.62, 54.90, 46.28, 45.40, 41.73, 38.51, 30.57, 23.86, 22.00, 21.63, 13.10, 12.99, 12.02, 11.90; HRMS (ESI+) Found 705.27872 Calculated. for C_34_H_41_N_8_O_7_S 704.28189 [M+H]+.

### Synthesis of (*S*‐1‐Methyl‐2‐nitro‐1*H*‐imidazol‐5‐yl)methyl {4‐methyl‐1‐[(3′*R*)−3′‐methyl‐4′‐(6′‐(1′‐(*S*‐methylsulfonimidoyl)cyclopropyl)−2′‐(1*H*‐pyrrolo[2,3‐*b*]pyridin‐4′‐yl)pyrimidin‐4′‐yl)morpholine]−1‐oxopentan‐2‐yl)carbamate (Nitroimidazolyl methyl carbamate‐Leu‐AZD6738, ICT10336) (7)

Leu‐AZD6738 (22 mg, 42 µmol) and DMAP (25 mg, 205 µmol) were added to a solution of **2** (27 mg, 84 µmol) in anhydrous CH_2_Cl_2_ (2.5 mL), and the reaction was stirred at RT overnight. Afterward, the volatiles were removed under reduced pressure, and the residue was purified by column chromatography on silica gel (CH_2_Cl_2_:MeOH 99:1→98:2→95:5) to afford compound **7** (21 mg, 71%) as a pale‐yellow solid. ^1^H NMR (400 MHz, DMSO‐*d*6) δ 11.81 (s, 1H, NH), 8.34 (d, 1H, *J*_3_ = 4.8 Hz), 7.89 (d, 1H, *J*_3_ = 4.8 Hz), 7.59 (m, 1H), 7.34 (d, 1H, *J*_3_ = 8.4 Hz), 7.17 (s, 1H), 6.75 (s, 1H), 5.08 (s, 2H), 4.62 (bs, 1H), 4.15 (m, 1H), 4.01 (dd, 1H, *J* = 2.4 Hz, *J* = 11.2 Hz), 3.86 (s, 3H), 3.80 (m, 2H), 3.64 (s, 3H), 3.59 (d, 1H, *J* = 9.2 Hz), 3.51 (ddd, 1H, *J* = 2.8 Hz, *J* = 11.6 Hz, *J* = 12.0 Hz), 3.27 (ddd, 1H, *J* = 3.6 Hz, *J* = 12.4 Hz, *J* = 13.6 Hz), 1.97 (m, 2H), 1.82 (m, 1H), 1.48 (m, 1H), 1.38 (m, 1H), 1.28 (d, 3H, *J* = 6.8 Hz), 1.14 (m, 1H), 0.97 (m, 1H), 0.86 (m, 1H), 0.64 (d, 3H, *J* = 6.4 Hz), 0.60 (d, 3H, *J* = 6.4 Hz); ^13^C NMR (100 MHz, DMSO‐*d*6) δ 180.39, 163.21, 162.47, 161.36, 155.69, 150.61, 146.42, 142.76, 137.11, 134.27, 128.77, 127.89, 118.14, 115.14, 102.55, 101.83, 70.67, 66.52, 55.92, 55.32, 46.95, 46.03, 41.18, 34.60, 31.43, 24.65, 23.27, 22.53, 21.53, 14.44, 14.11, 13.84; HRMS (ESI+) Found 709.28664 Calculated for C_32_H_41_N_10_O_7_S 709.28804 [M+H]^+^.

### Synthesis of (*S*)‐Benzyl {4‐methyl‐1‐[(3′*R*)−3′‐methyl‐4′‐(6′‐(1′‐(*S*‐methylsulfonimidoyl)cyclopropyl)−2′‐(1*H*‐pyrrolo[2,3‐*b*]pyridin‐4′‐yl)pyrimidin‐4′‐yl)morpholine]−1‐oxopentan‐2‐yl}carbamate (Benzyl carbamate‐Leu‐AZD6738, ICT10337) (8)

Leu‐AZD6738 (17 mg, 32 µmol) and 4‐methylaminopyridine (DMAP, 19 mg, 153 µmol) were added to a solution of **3** (18 mg, 64 µmol) in anhydrous CH_2_Cl_2_ (1.9 mL), and the reaction was stirred at RT overnight. Afterward, the volatile were removed under reduced pressure, and the residue was purified by column chromatography on silica gel (DCM:MeOH 99:1→98:2→95:5) to afford titled compound **8** (15 mg, 71%) as a white solid. ^1^H NMR (400 MHz, CDCl_3_) δ 9.39 (s, 1H, NH), 8.42 (d, 1H, *J*_3_ = 4.8 Hz), 8.02 (d, 1H, *J*_3_ = 4.8 Hz), 7.44 (dd, 1H, *J*_H,NH_ = 2.6 Hz, *J*_3_ = 3.4 Hz), 7.31 (dd, 1H, *J*_H,NH_ = 2.2 Hz, *J*_3_ = 3.4 Hz), 7.26 (m, 5H), 6.76 (s, 1H), 5.24 (d, 1H, *J* = 8.8 Hz), 5.03 (d, 1H, *J*_2_ = 12.4 Hz), 4.98 (d, 1H, *J*_2_ = 12.4 Hz), 4.58 (m, 1H), 4.21 (m, 1H), 4.06 (dd, 1H, *J* = 3.6 Hz, *J* = 11.6 Hz), 3.84 (d, 1H, *J* = 11.6 Hz), 3.75 (dd, 1H, *J* = 2.8 Hz, *J* = 11.6 Hz), 3.63 (ddd, 1H, *J* = 2.8 Hz, *J* = 11.6 Hz, *J* = 12.4 Hz), 3.49 (s, 3H), 3.38 (ddd, 1H, *J* = 3.6 Hz, *J* = 11.6 Hz, *J* = 12.4 Hz), 2.18 (m, 1H), 1.90 (m, 1H), 1.71 (m, 1H), 1.67‐1.55 (m, 2H), 1.48 (m, 1H), 1.39 (d, 3H, *J* = 6.4 Hz), 1.25 (m, 2H), 0.88 (d, 3H, *J* = 3.6 Hz), 0.87 (d, 3H, *J* = 4.8 Hz); ^13^C NMR (100 MHz, CDCl_3_) δ 181.13, 163.74, 162.32, 160.55, 155.87, 150.17, 143.04, 137.67, 136.47, 128.44 (2C), 128.01, 127.96 (2C), 125.93, 115.74, 103.34, 102.92, 70.93, 66.67, 66.61, 55.85, 47.28, 46.44, 42.90, 40.27, 39.54, 31.60, 24.85, 23.02, 22.67, 13.91, 13.12, 12.82; HRMS (ESI+) Found 660.29458 Calculated for C_34_H_42_N_7_O_5_S 660.29681 [M+H]^+^.

## Conflict of Interest

The authors declare no conflict of interest.

## Author Contributions

F.M.B. performed conceptualization, methodology, investigation, validation, funding acquisition, wrote the original draft preparation and review. G.R.M. performed investigation – Compound synthesis and characterization, and contributed to original draft preparation. P.M.L. performed validation, reviewed and edited the draft. R.A.F. performed methodology, validation, reviewed and edited the draft, and provided resources. S.F.E. performed methodology, funding acquisition, validation, reviewed and edited the draft, and provided resources. Final proof‐reading was done by all authors.

## Supporting information

Supporting Information

## Data Availability

All data reported in this manuscript are available from the lead contact without restriction. Original western blot images have been deposited at Mendeley [https://doi.org/10.17632/5j36vswdfm.1] and are publicly available as of the date of publication.
