# Supplementary material for: Hypoxia‐Responsive Prodrug of ATR Inhibitor, AZD6738, Selectively Eradicates Treatment‐Resistant Cancer Cells
Source: Adv Sci (Weinh). 2024 Jul 8;11(34):2403831. doi: 10.1002/advs.202403831 (PMC11425890; doi:10.1002/advs.202403831)
Supplement: Supplementary file 1 — Supporting Information [file ADVS-11-2403831-s001.docx]

**Hypoxia-Responsive Prodrug of ATR Inhibitor, AZD6738, Selectively Eradicates Treatment-Resistant Cancer Cells**

Francis M. Barnieh^1^*, Goreti Ribeiro Morais^1^, Paul M. Loadman^1^, Robert A. Falconer^1^, Sherif F. El Khamisy^1,2^

^1^Institute of Cancer Therapeutics, Faculty of Life Sciences, University of Bradford, Bradford, UK.

^2^School of Biosciences, University of Sheffield, Sheffield, UK.

***Correspondence:** [f.mprahbarnieh1@bradford.ac.uk](mailto:f.mprahbarnieh1@bradford.ac.uk)

**Supplementary Information**

**AZD6738**

**DMSO**

**1.0 µM**

**Hypoxia**

**Normoxia**


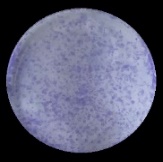

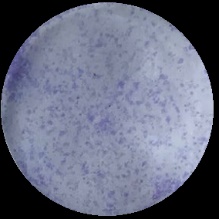

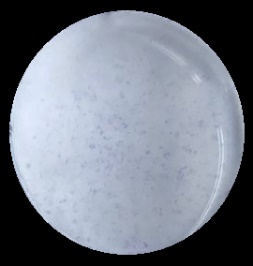

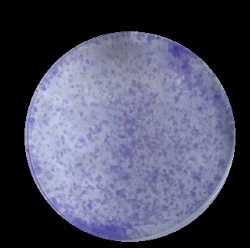


**MDA-MB-231**


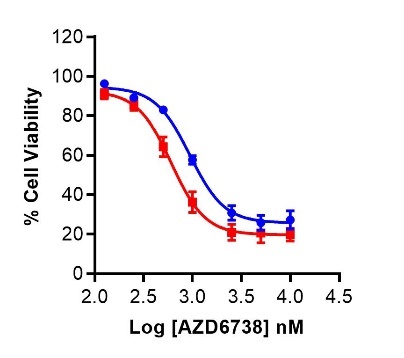

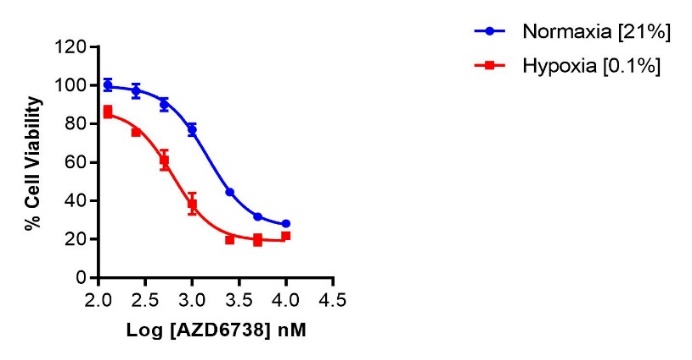


**MDA-MB-231**

**MDA-MB-468**


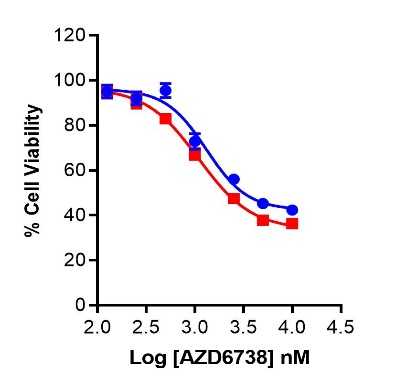


**HS578T**

**AZD6738**

**µM**

**N**

**=**

**H**

**=**

**1.0**

**0**

**1.0**

**0**

**HS578T**

- **p-H2AX**

- **Actin**


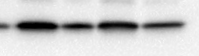


15 -


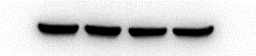


37-

100 -

- **HIF-1A**


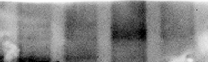


- **ATR**


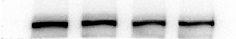


250 -

**A**

**B**

**C**


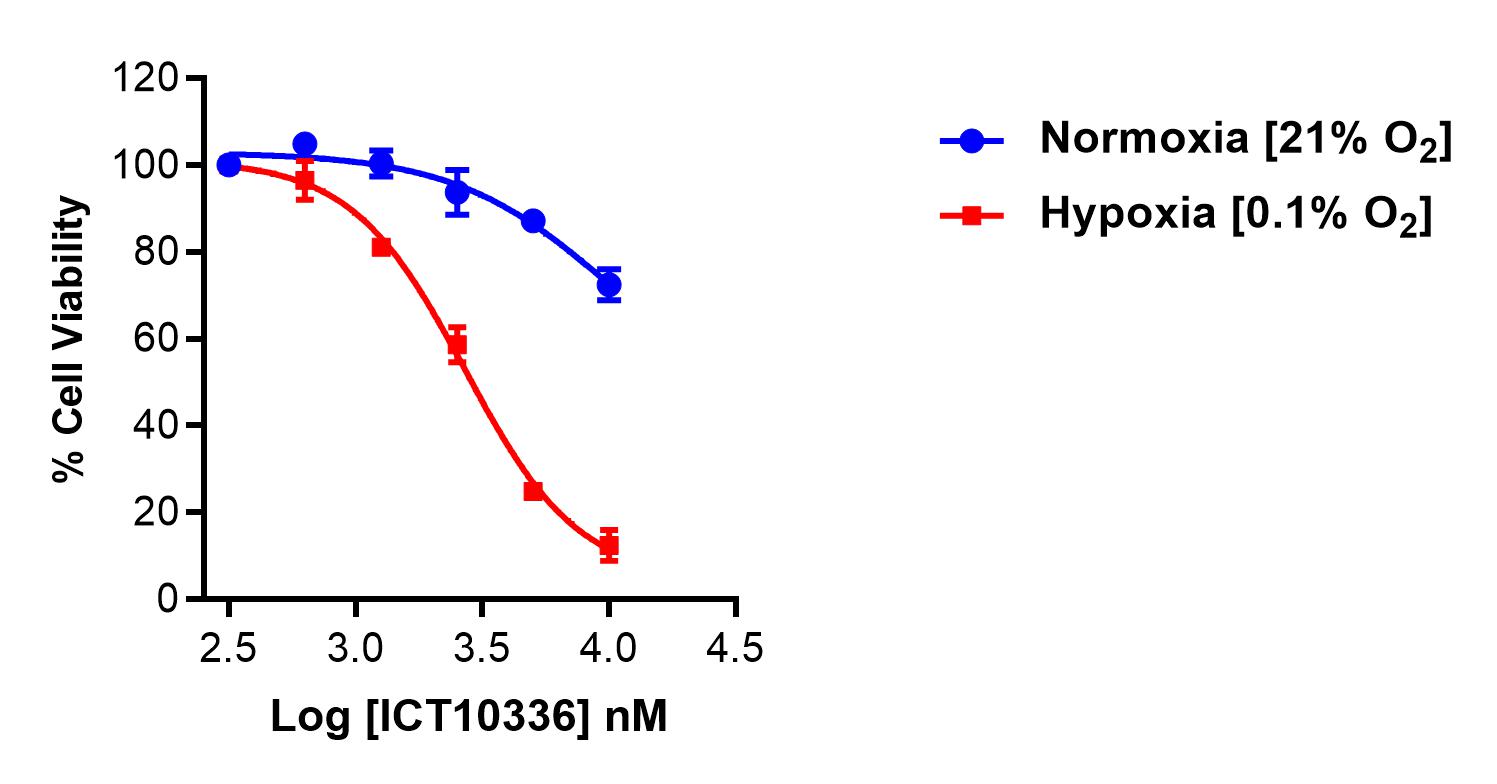


**Fig S1:** Hypoxia sensitises TNBC cells to AZD6738 treatment. (A) TNBC cells were treated with various concentrations of AZD6738 in normoxic and hypoxic conditions, for 24 h and regrow in drug-free medium in normoxic conditions for 72 h. Cell viability was assessed via an MTT assay. (B) Clonogenic assay results of MDA-MB-231 cells after exposure to AZD6738 for 24 hours in normoxic and hypoxic conditions and allow to regrow in regrowth in drug-free medium in normoxic conditions for 12 days. (C) HS578T cells treated with AZD6738 (1 µM) for 24 hours in either normoxic (N) or hypoxic (H) conditions and cell lysates were analysed by immunoblotting for the indicated proteins. (C&D) (D). Data shown are the mean of ≥ 3 independent experiments ± SEM.


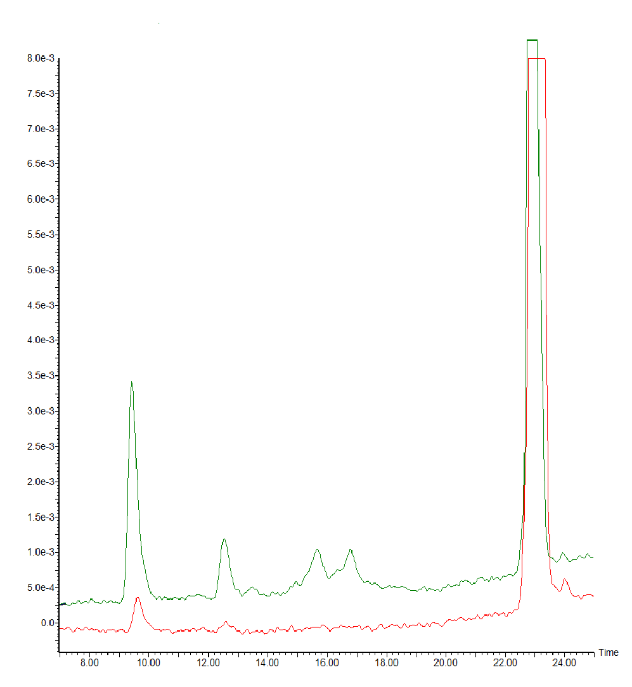


**ICT10336**

**AZD6738**

**Normoxia**

**Hypoxia**

**Leu-AZD6738**

**Retention Time (mins)**


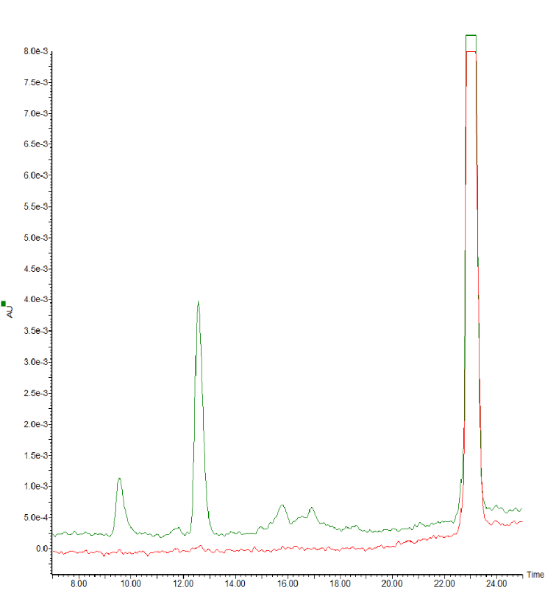


**Normoxia**

**Hypoxia**

**ICT10336**

**AZD6738**

**Leu-AZD6738**

**Retention Time (mins)**

**A**

**B**

**Fig S2:** *Ex-vivo* activation and release of AZD6738 and metabolites from ICT10336. Representative of LC-MS spectra demonstrating differential metabolism of ICT10336 in normoxic and hypoxic human cancer CDX homogenates; MCF-7 CDX (A) and DLD-1 CDX (B), after 1-hour of incubation.

**MDA-MB-231**


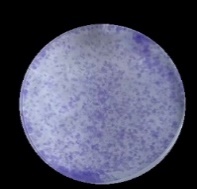

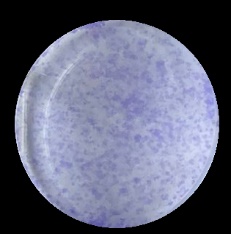

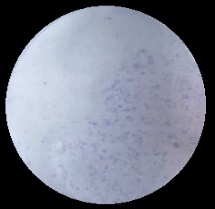

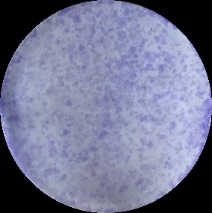


**ICT10336**

**DMSO**

**5.0 µM**

**Normoxia**

**Hypoxia**

**N**

**H**

**N**

**H**

**N**

**H**


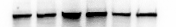

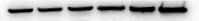


- **CYPOR**

- **Actin**

100 -

37 -

**MDA-MB-231**

**MDA-MB-468**

**HS578T**


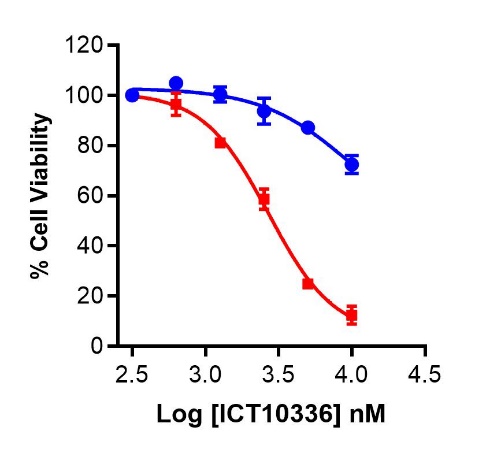

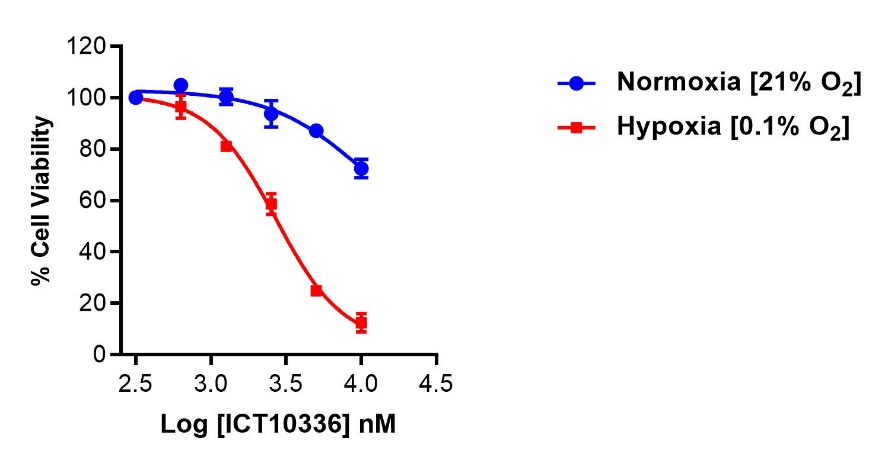


**HS578T**

**IC_50_ > 10.0 µM 0.15**

**IC_50_ = 2.81 ± 0.15 µM**

**A**

**B**

**C**

**Fig S3:** Anti-proliferative activity of ICT10336 is hypoxia-dependent. (A) CYPOR protein expression in TNBC cells lines incubated in either normoxic condition (N) or hypoxic condition for 24 hours. (B) Hs578T cells were treated with various concentrations of ICT10336 in normoxic and hypoxic conditions for 96 hours. Cell viability was assessed via an MTT assay. (C) Clonogenic assay results of MDA-MB-231 cells after exposure to ICT10336 for 24 hours in normoxic and hypoxic conditions and allow to regrow in regrowth in drug-free medium in normoxic conditions for 12 days

**Figure S4**. Structure of 4-nitrophenyl carbonate intermediates **1-3** related to scheme 2.

**Table S1**: IC_50_ values of AZD6738 and ICT10336 in normal healthy cells) after 24-hours treatment and 72-h regrowth in drug-free medium in normoxic conditions

| Normal Cells | IC_50_ (µM) | | PSI |
| --- | --- | --- | --- |
|  | AZD6738 | ICT10336 |  |
| HEK293T | 0.89 ± 0.03 | >10 | **>11.2** |
| MRC-5 | 1.27 ± 0.05 | 8.82 ± 0.62 | **6.9** |

*Prodrug Safety Index (PSI) is the ratio of the cytotoxicity (IC_50_) of the prodrug (ICT10336) to the cytotoxicity (IC_50_) of the warhead (AZD6738) in normal cells.*
